# Assessing the short-run effects of lockdown policies on economic activity, with an application to the Santiago Metropolitan Region, Chile

**DOI:** 10.1371/journal.pone.0252938

**Published:** 2021-06-21

**Authors:** Constanza Fosco, Felipe Zurita

**Affiliations:** 1 Research Center for Integrated Disaster Risk Management (CIGIDEN), ANID/FONDAP/15110017, Santiago, Chile; 2 Grupo Interdisciplinar de Sistemas Complejos (GISC), Spain; 3 Instituto de Economía, Pontificia Universidad Católica de Chile, Santiago, Chile; Nanyang Technological University, SINGAPORE

## Abstract

This paper develops a methodology for the assessment of the short-run effects of lockdown policies on economic activity. The methodology combines labor market data with simulation of an agent-based model. We apply our methodology to the Santiago Metropolitan Region, Chile. We recover the model parameters from observed data, taking into account the recurring policy adjustments that characterized the study window. The model is used to build counterfactual scenarios. We estimate an 8 percent output loss in the first 5 months of the pandemic from the policy that was put in place, achieving a 56 percent reduction in the total number of infections. During this period, with an output loss to 10.5 percent of GDP, the infection rate would have decreased 92 percent, significantly delaying the spread of COVID and spike in infections. Our methodology applied to real data provided results that could be valuable in guiding policies in other lockdown situations in times of disaster, pandemics or social upheaval.

## 1 Introduction

This paper develops a methodology for assessing the short-run economic effects of an epidemic and the ensuing health policy response. In particular, we estimate the labor, income, health, and welfare consequences of lockdown policies during the first five months of the COVID-19 epidemic in the Santiago Metropolitan Region (henceforth, SMR), Chile. The methodology combines labor survey data with simulation of a meta-population, compartmental SIR (Susceptible-Infected-Removed) model.

The aim of public policy in an epidemic is to minimize the death toll, with the least possible sacrifice in other dimensions. Chief among those concerns is avoiding the loss of jobs and livelihoods. At the onset of the epidemic this is best achieved through tracing and testing [1]. However, when the disease is already spread beyond traceability, the next best solution seems to be locking down enough areas so that the public health system can cope with the diseased. A lockdown might be effective, but has an obvious downside: many people are prevented from working. The immediate consequences include output loss, income loss for the most vulnerable workers (mainly the self-employed and the informal workers), and decreased consumption for those people lacking savings, insurance, or out-of-reach from governmental relief programs. This is the type of disaster in which the design of better policy responses can contribute a great deal to contain the welfare loss.

The spatial dimension is essential to the phenomenon at hand, for three reasons. First, the disease spreads locally, through contact. Second, in a segregated city, most worker types are clustered in particular areas. Neighborhoods with a large fraction of essential workers (i.e., those that, by the nature of their activity, the authorities deem necessary or even mandatory that they keep working under lockdown; the activities include medical services, food and medicine production and distribution, utilities, policing, etc. The precise definition varies from country to country; S2. Table in S1 File explains the definition adopted by the Chilean authorities) may not reduce mobility. At the other extreme, in neighborhoods where most workers can work from home, mobility could be severely reduced with or without a lockdown, and without significant economic costs. Hence, the ability of the lockdown to affect mobility and its economic costs depends on the local composition of the labor force. Third, the travel patterns within a city will determine the spatial trajectory of the disease, at least in the early phases. Since work-related travel is a large fraction of total travel in normal times, and much more so under lockdown, it is bound to be determinant to the disease spread.

**Fig 1:**
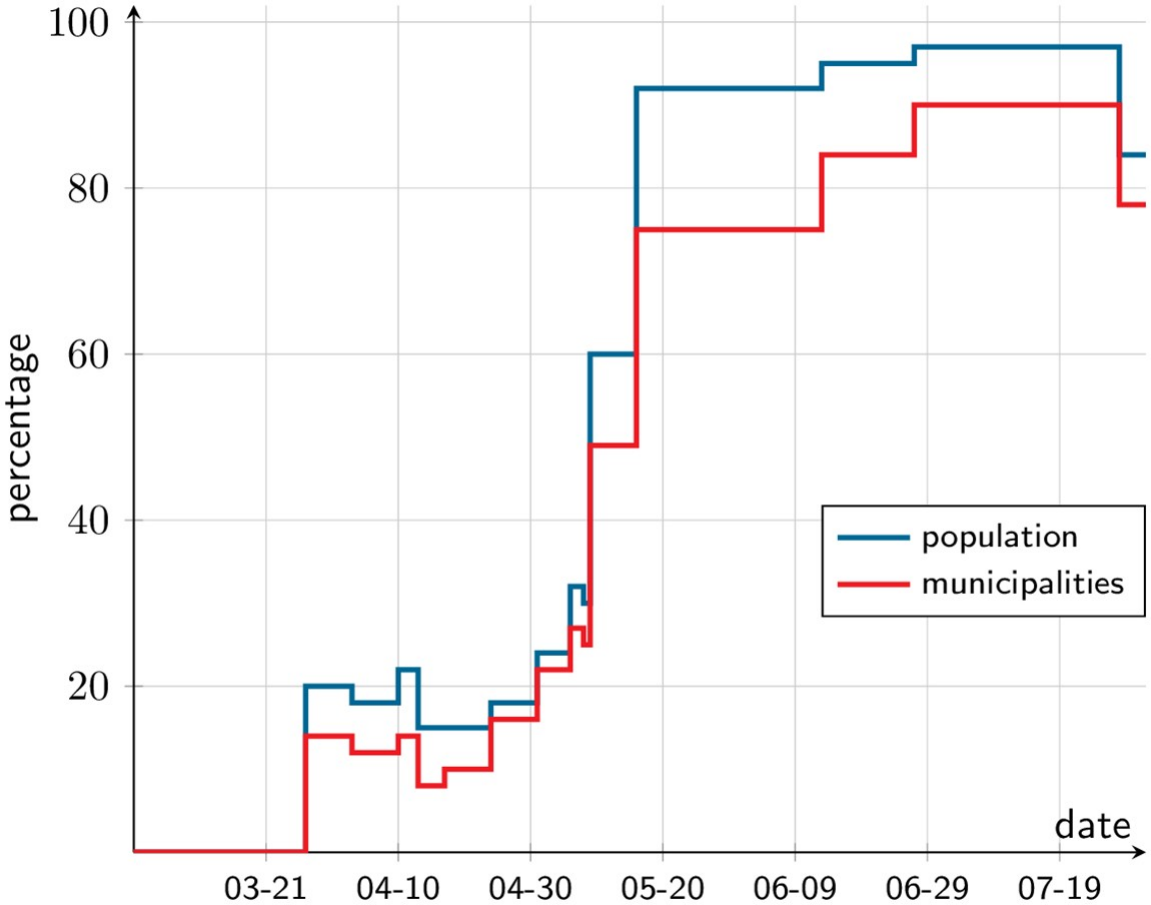
Chile’s targeted lockdown policy. Percentage of inhabitants (blue) and municipalities (red) under lockdown in the SMR. From March 1 till August 1, 2020.

We apply our methodology to the SMR. Studying this Chilean metropolitan area is interesting on a number of counts. First, Chile is a middle-income country and, as such, its experience may shed light on the particularities of this type of country; most such studies focus on advanced economies. Second, the epidemic arrived two months after it was found in Europe, but it spread with particular intensity: as of July, Chile was among the top ten countries in the world in terms of per capita infections. Third, Santiago is a highly segregated metropolis [2–4]. Segregation has implications for the contagion process as well as for economic activity. Lastly, the government followed a policy of targeted lockdowns, by which municipalities were put under and out of lockdown on a regular basis. Fig 1 illustrates the percentage of municipalities and the percentage of inhabitants under lockdown during the first five months of the epidemic, which is our estimation window; S5 Table in S1 File lists the implemented measures. Crucially, the constant change in restrictions makes recovering the contagion-process parameters from observed data particularly challenging. Our proposal is to take advantage of the flexibility that the simulation approach provides, and incorporate as much detail regarding the policy variations over time and space as possible, to better infer those parameters.

The epidemic is thus modeled by means of a data-driven, agent-based metapopulation SIR model as per [5–7]. (See [8] for a review of data-driven, agent-based models, and [9–11] for specific applications. [12] summarizes this literature as applied to the COVID-19 epidemic.) Each subpopulation lives in a municipality, and the connection between any two subpopulations is given by the (possibly bidirectional) commuting flow. This simple spatial structure allows us to consider pre-pandemic heterogeneity and, fundamentally, the targeted lockdown strategy. At every moment, the currently present agents at each municipality are assumed to be well-mixed. Metapopulation data-driven models have been profusely applied to assess the impacts of non-pharmaceutical interventions on the COVID-19 epidemic on specific countries or regions, but most do not consider economic activity. For instance, studies of Spain [13], Northeast Ohio and Southeast Michigan [14], Belgium [15], Brazil [16], France [17], and Italy [18].

We work with real data (scaled 1:15) with an exhaustive description of the worker characteristics, and we simulate the model in a stochastic and individual-mechanistic fashion. We consider each agent’s situation at different moments within the day, explicitly move commuters between municipalities, and consider whether they are able to move or work remotely when they are under some confinement rule.

The model is used to produce counterfactual scenarios. Our interest lies in three variables: health status of the population, total output loss, and a measure of material welfare. Our results show that in the absence of any lockdown policy, the disease spreads quickly, and herd immunity is reached just within four months, with about 70 percent of the population infected. Under the policy the authorities followed, cumulative infections were 56 percent lower by August 1, 2020, than under no lockdown. Under full-lockdown, cumulative infections would have been 80 percent less than what they actually were, or 92 percent less than under no lockdown. These estimates cast lockdowns as very effective.

Regarding the economic cost of these policies, we compare output in different scenarios against a baseline situation, defined as the scenario without epidemic. Output is estimated to be 8 percent smaller than the baseline by August 1, 2020, in the actual scenario with the epidemic, and the way it was handled. The more stringent lockdown would have added 2.5 additional points, but with a large health benefit. A complete evaluation of the alternative policy options should consider the full duration of the event. We do not attempt such analysis because of the many uncertainties surrounding the epidemic (e.g., the development, distribution, and effectiveness of the vaccines, and the array of strands of the virus that are likely to emerge), and because our model is intended to capture only short-term economic effects (on this regard, see the discussion of limitations below). Still, we can take it as a first approximation that a one-year-long full lockdown would cost as much as 30 percent of GDP. But even in the presence of an unforeseeable future, delaying the spread of the disease should be considered a gain.

In the spatial dimension, we observe large differences in labor composition among municipalities that translate directly into varying reactions to lockdowns. This is true with regards to job essentiality, the ability to work from home, and whether pay is contingent upon showing up to work. Also, the fact that municipalities are interconnected through commuting implies that there are externalities among them: locking down one municipality might have even larger effects on another than on itself, in variables such as employment, income, and disease spread. Finally, the analysis shows a remarkable relationship between the output cost of locking down one municipality, and its eigenvector centrality, a graph-theoretical measure of node importance, as constructed from the commuting origin-destination matrix.

Our model is rich in the spatial labor composition and disease tracking dimensions, which seem critical for the recovery of the contagion parameters. It simplifies in others. Importantly, it may only look at the direct, short-run, economic consequences. In particular, the production model is additive, as in [19]. This means that it doesn’t take into account production interdependencies among industries. Neither does it consider other general equilibrium effects, such as price adjustments, innovations, or technology adaptations that are likely to occur in time. Indeed, while some job vacancies are destroyed, others are created, as people cope with the new conditions; such dynamics are absent from our model. Therefore, evaluations concerning periods longer than a few months should be considered as first-order approximations.

In the span of less than a year, an array of models have been produced that place emphasis on different questions and employ a variety of methodologies. We don’t attempt a full review, but rather, we make some remarks where pertinent.

Within agent-based metapopulation epidemic models, [20, 21] stand out. They both look for optimal targeted lockdowns; the former allows for a proportional reduction in the overall interaction within each location, and the latter allows for spatial targeting. They both conclude that the optimal lockdown policy is not uniform. Although we don’t look for optimal policies, we find that spatial heterogeneity in essentiality and teleworkability impose constraints on such policies. Thus, we provide an empirical framework against which the feasibility of an optimal targeted lockdown can be evaluated.

The largest class of economic papers on the epidemic builds over dynamic macroeconomic models, and extends them to incorporate a compartmental epidemic model, usually in the form of a set of differential equations. The main advantage of this approach is that it is suited to analyzing socially optimal outcomes, from a social planner or utilitarian perspective, as well as competitive equilibrium. [22] emphasize that equilibrium is not socially optimal, because the contagion process introduces externalities. Social optimality requires containment policies, at the expense of a more severe economic recession. Similarly, [23] find that the optimal reduction of social and economic activity is preventive and smoother than the equilibrium reduction because, in the latter, the reduction obtains only when there are already many infected cases.

Among the many modelling choices, that of control variable(s) is particularly relevant. [19] cast the planner’s problem to choose the fraction of the population that is subject to lockdown at each moment in time. In contrast, [24] consider a two-dimensional policy: the fraction of activity in a luxury sector to shut down (mitigation policy), and how much income to redistribute from those who can work to the rest of the population (transfer policy). [25–27] consider multi-group risk epidemic models, grouping individuals by age and/or economic activity. And finally, [28] study business protocols as a way of reducing contagion while avoiding production shut down, allowing for heterogeneity across economic sectors or industries. A general finding in this literature is that differentiated containment policies are better than uniform ones.

A few examples of further methodologies include: [29], who used value added tax data by municipality to econometrically measure the ex post effect of the targeted lockdown policy in Chile. Their main findings are that lockdowns produced on average a 10–15% drop in local economic activity until May 2020, and that the economic costs are proportional to the population under lockdown. [30] resorted to an input-output model to study the regional situation in Germany. Finally, [31] estimated a vector autorregresive model using data on historical disasters in the USA.

The rest of the paper is organized as follows. Section 2 describes the model. Section 3 describes the model’s implementation in the SMR, starting from a characterization of the pre-COVID baseline situation, and the calibration per se. Section 4 presents the main results. Section 5 concludes. The S1 and S2 Appendices in S1 File presents the definition of the variables and the description of the simulation model.

## 2 The model

The framework is an agent-based model with a metapopulation spatial structure. A geographical area is partitioned into municipalities, indexed by *j* = 1, …, *m*. Time is indexed by *t* = 0, 1, 2, …; the basic time unit is a day, but days are further divided into three intervals *s* = 1, 2, 3, or shifts: morning, afternoon, and evening. At every day *t*, each municipality *j* may be under lockdown ($q_{jt}=1$), or not ($q_{jt}=0$). Lockdowns that only affect some areas within a municipality can be accommodated by assuming $q_{jt}\in \left[0,1\right]$, with the interpretation that a person that works or lives at municipality *j* has a probability $q_{jt}$ of being confined.

There is a finite set of individuals, indexed by *i* = 1, …, *n*. Each individual (she/her) is defined by a time-independent vector of variables $x_{i}=\langle a_{i},b_{i},w_{i},w_{i},e_{i},z_{i},c_{i}\rangle$, and her evolution characterized by a time-dependent state vector $y_{its}=\langle s_{it},i_{it},r_{it},d_{it},l_{it},f_{its},m_{it},\kappa_{its}\rangle$. The characteristics include her municipality of residence, *a*_*i*_; her municipality of work, *b*_*i*_; her daily wage if employed *w*_*i*_; the indicator variables $w_{i}$, whether she is employed or not; $e_{i}$, if employed, whether she works in an essential sector or not; $z_{i}$, whether her work can be done remotely or she must work on-site; and $c_{i}$, whether or not she would keep her wage even if she couldn’t show up at work. The state vector contains the indicator functions $s_{it}$, $i_{it}$, $r_{it}$, and $d_{it}$ –whether she is susceptible, infected, recovered, or dead, respectively, at day *t*–; $l_{it}$, whether she is isolated o not; $f_{its}$, whether or not she has a shift at day segment *s* at day *t*; and the stringency of her confinement, *κ*_*its*_–to be explained shortly. Naturally,

$$\begin{array}{cc} \left(\forall i,t\right)\;s_{it}+i_{it}+r_{it}+d_{it} & =1, \end{array}$$ (1)

meaning every person is in exactly one health state at every day.

A person goes to work or is mobile at day *t*, denoted by the indicator variable $m_{it}$, if she has a job ($w_{i}=1$), she is not dead nor isolated ($d_{it}+l_{it}=0$), and: (i) if under confinement (either her home or her workplace municipalities are under lockdown, $q_{a_{i}t}+q_{b_{i}t}-q_{a_{i}t}q_{b_{i}t}=1$), she cannot telework ($z_{i}=0$) and her job is essential ($e_{i}=1$); or (ii) if not under confinement (neither her home nor her workplace municipalities are under lockdown, $q_{a_{i}t}+q_{b_{i}t}-q_{a_{i}t}q_{b_{i}t}=0$):

$$\begin{array}{c} m_{it}\equiv w_{i}\left(1-d_{it}-l_{it}\right)(\left(q_{a_{i}t}+q_{b_{i}t}-q_{a_{i}t}q_{b_{i}t}\right)\left(1-z_{i}\right)e_{i}+\left(1-q_{a_{i}t}-q_{b_{i}t}+q_{a_{i}t}q_{b_{i}t}\right)). \end{array}$$ (2)

If a full-time employed worker works, she does so the first two day segments or shifts ($f_{its}=1$ for *s* = 1, 2). Part-time employed workers do it either in the morning or the afternoon shift; we assume that half of the part-time workers work at each shift. The unemployed, the retired and the underage spend the whole time at their home municipality. At each day segment (*t*, *s*), each individual *i* is at exactly one municipality: the municipality of workplace if working on-site, and the home municipality otherwise. Let *p*_*its*_ be the municipality where *i* is present at (*t*, *s*), i.e.,

$$\begin{array}{c} p_{its}\equiv \left(1-m_{it}f_{its}\right)a_{i}+m_{it}f_{its}b_{i}. \end{array}$$ (3)

The municipality-wide aggregates of the dummy variables in (**x**_*i*_,**y**_*its*_) are labeled as their regular-font counterparts, namely, {*s*_*jt*_, *i*_*jt*_, *r*_*jt*_, *d*_*jt*_, *l*_*jt*_, *e*_*jt*_, *z*_*jt*_, *c*_*jt*_, *f*_*jt*_}_*j* ∈ *J*_ when considering the municipality of residence, and with an upper bar when considering the municipality where the person is at day time-segment (*t*, *s*), ${\left\{{\bar{s}}_{jts},{\bar{i}}_{jts},{\bar{r}}_{jts},{\bar{d}}_{jts},{\bar{l}}_{jts},{\bar{e}}_{jts},{\bar{z}}_{jts},{\bar{c}}_{jts},{\bar{f}}_{jts}\right\}}_{j\in J}$. For instance, the number of susceptible individuals at municipality *j* at day *t* is the number of susceptible residents at *j*:

$$\begin{array}{cc} s_{jt}\equiv & \sum_{\left\{i\in I:a_{i}=j\right\}}s_{it}, \end{array}$$ (4)

while the number of susceptible individuals present at municipality *j* at shift *s* is given by

$$\begin{array}{cc} {\bar{s}}_{jts}\equiv & \sum_{\left\{i\in I:a_{i}=j\right\}}s_{its}\left(1-m_{it}f_{its}\right)+\sum_{\left\{i\in I:b_{i}=j\right\}}s_{its}m_{it}f_{its}, \end{array}$$ (5)

meaning that people are counted at their municipality of residence if not working or teleworking at a particular shift, and at their municipality of work otherwise. The rest of the variables are defined likewise.

### 2.1 Contagion

We adapt the modeling of contagion from [32, 33], and follow [34] in making the distinction between “cities” and “small villages”. Cities are population-dense and highly interconnected areas; small villages are neither. The contagion probability will depend on the number of present individuals in the former, but not on the latter.

Within each municipality, non-isolated individuals are assumed to be well-mixed. Recalling that *p*_*its*_ is the municipality where *i* is present at day-segment (*t*, *s*), and *n*_*p*_*its*_ *ts*_ and *i*_*p*_*its*_ *ts*_ are the total number of persons present and infected at that municipality, respectively, then a susceptible, non isolated, agent gets infected with probability

$$\begin{array}{c} \pi_{it}\equiv \mathrm{Pr}\left(i_{i,t}=1|s_{i,t-1}=1\right)\approx \sum_{s=1}^{3}(1-{\left(1-\frac{\beta }{n_{its}^{*}}\tau_{s}\kappa_{its}\right)}^{i_{p_{its}ts}}), \end{array}$$ (6)

where

$$\begin{array}{c} n_{its}^{*}=\{\begin{array}{cc} n_{p_{its}ts} & \;\text{if}\;p_{its}\;\text{is}\;\text{a}\;\text{city,}\;\text{and}\\ n_{0} & \text{if}\;p_{its}\;\text{is}\;\text{a}\;\text{small}\;\text{village}, \end{array} \end{array}$$

and *β* is the contagion rate –a disease parameter–; *τ*_*s*_ is the length of day segment *s*; and *κ*_*its*_ is a behavioral exposure variable reflecting the effect of confinement and self care. There are three possible degrees of confinement, *κ*_1_, *κ*_2_, and *κ*_3_, where:

$$\begin{array}{c} \kappa_{its}=\{\begin{array}{cc} \kappa_{3}=1 & \text{if}\;\text{not}\;\text{confined,}\\ \kappa_{2} & \text{if}\;\text{under}\;\text{soft}\;\text{confinement,}\\ \kappa_{1} & \text{if}\;\text{under}\;\text{strict}\;\text{confinement,} \end{array} \end{array}$$ (7)

and where 0 < *κ*_1_ < *κ*_2_ < 1. For simplicity, Eq 6 considers negligible the probability of an individual getting infected twice or three times during the day. This is to say, the health state is updated only at the end of each day.

All individual contagion probabilities {*π*_*it*_} are mutually independent. According to Eq 6, a person’s contagion probability is assumed to increase with the number of present, infected people (*i*_*p*_*its*_ *ts*_), the disease contagion rate (*β*), and the length of exposure (*τ*_*s*_); and to decrease with the number of people present (*n*_*p*_*its*_ *ts*_) in the case of cities, and the degree of confinement the person adopts (*κ*_*its*_). Thus, each individual is subject to a different risk depending on the characteristics and state of the municipalities where she spends her day.

**Fig 2:**
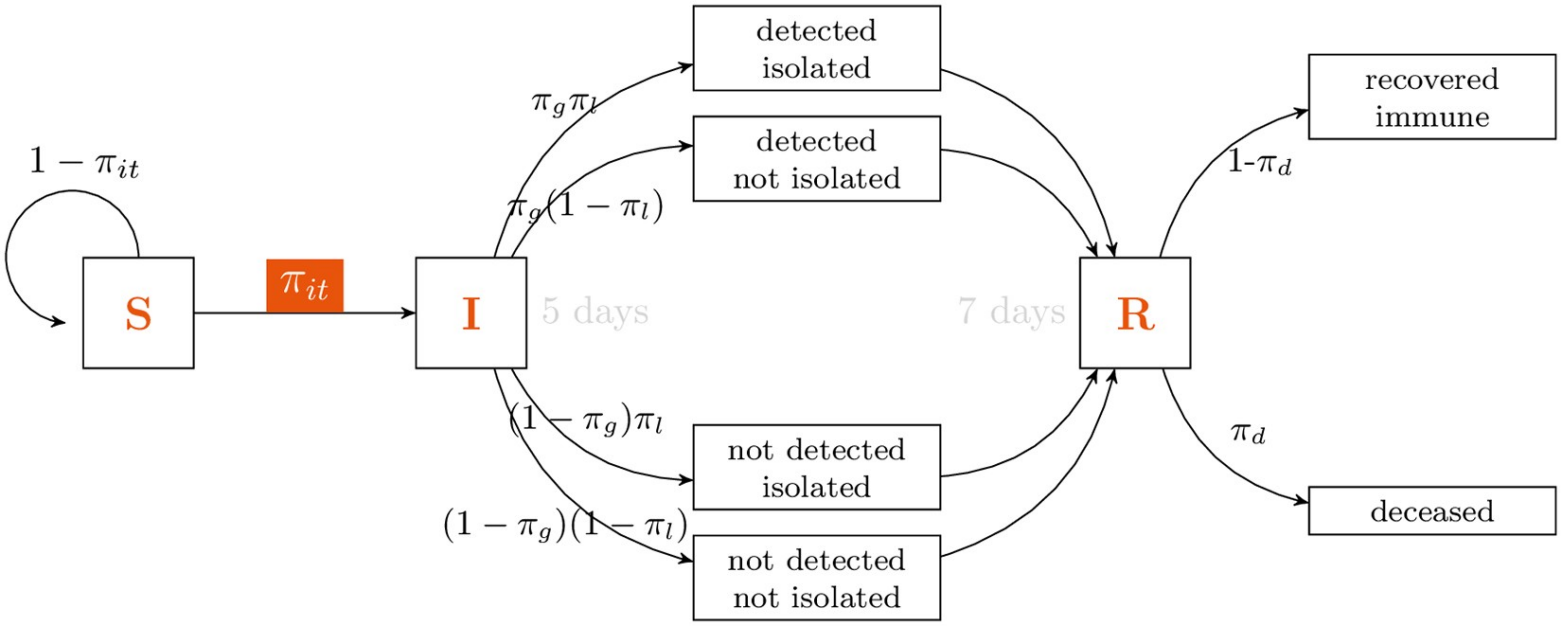
The SIR model. Succeptible individuals become Infected with probability *π*_*it*_: infected individuals may be detected and/or isolated, after which they are Removed (meaning they recover or die).

The individual health status is governed by a Markov process with the following daily transition matrix (oriented column to row):

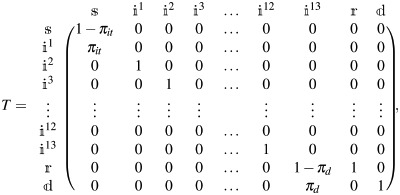
(8)
where the variable $i^{k}$ takes the value 1 if the person was infected *k* days ago, and 0 otherwise. This process is further illustrated in Fig 2. Susceptible individuals thus have a probability *π*_*it*_ of catching the disease, which is sensitive to the conditions of the municipality they are in (Eq 6). Once infected, the model assumes that disease cannot be detected during the first five days; yet, the individual is spreading the disease since the first day. At the beginning of the sixth day, sick patients are sorted between detected (with probability *π*_*g*_) and not detected, and between isolated (with probability *π*_*l*_) and not isolated. We assume for simplicity that these processes are independent. Patients remain sick for another seven days. Twelve days after contagion (i.e., at the beginning of the thirteenth day), the individual becomes either recovered (and immune afterwards), or dead. The latter, with probability *π*_*d*_–the infection fatality ratio (IFR).

### 2.2 Labor market

The population is characterized by the distribution of **x**_*i*_ across municipalities. Consumption is assumed to take place at the municipality of residence. Geography is included by considering municipalities as local neighborhoods, and their interconnections by commuting patterns present in the baseline scenario.

The only communicating vessels among municipalities are commuting workers. Their inclusion in the model is a key feature, as we seek to understand the interaction of the disease with the job market. On the other hand, considering only commuting workers implies that the pattern of mobility is recurrent, with fixed origin- and destination-subpopulations [35–37].

The commuting origin-destination (OD) matrix **M**_*t*_ has as its (*j*, *k*) entry

$$\begin{array}{c} m_{jk}^{t}\equiv \sum_{\left\{i:\;a_{i}=j\wedge b_{i}=k\right\}}m_{it}, \end{array}$$ (9)

namely, the number of workers that reside at *j* and go to work at *k* in day *t*. **M**_0_ reflects the initial situation, without any lockdown; different lockdown policies would produce different OD matrices each day, {**M**_*t*_}, where **M**_*t*_ ≤ **M**_0_, as is clear from Eq 2.

There are two conditions that are particularly relevant for the co-evolution of economic activity and the disease: the ability to work from home ($z_{i}$), and the character of “essential worker” that a job may or may not possess ($e_{i}$). On the other hand, worker welfare depends heavily on wage security, namely, having the tranquility of receiving pay even if unable to work ($c_{i}$). The combination of these variables produces four types of worker:

I. on-site, essential workers ($\left(1-z_{i}\right)e_{i}=1$);
II. on-site, non essential workers ($\left(1-z_{i}\right)\left(1-e_{i}\right)=1$);
  - secure pay, or unconditional ($c_{i}=0$);
  - insecure pay, or conditional ($c_{i}=1$);
III. off-site, remote, or tele-workers ($z_{i}=1$).

Type-I workers will have to leave home even under a stringent lockdown. The size of this group then sets a lower bound on mobility for the municipalities involved. Type-III workers could stay at home and keep working even without a lockdown, setting an upper bound on mobility. Thus, type-II workers are the lockdown-sensitive group: They are actually deprived of their right to work during a lockdown. In this group, some workers’ pay is protected by contracts or employer will, i.e., their wage is unconditional (type IIa). Others, mostly the informal workers, the self-employed, and those under short-term contracts lose their income during a lockdown (type IIb) because their pay is conditional on coming to work and working.

### 2.3 Lockdowns

We consider the possibility that a lockdown is decreed for a subset of municipalities. This is the case in Chile, where a targeted lockdown policy is in place. Let *Q*_*t*_ ⊆ *J* be the set of municipalities under quarantine at day *t*.

We track a few key concepts, namely, the infected population, output loss, and welfare, and compare them to the baseline situation (i.e., at day *t* = 0). We discuss them briefly below.

#### 2.3.1 Cases

The total number of cases by municipality, i.e., the cumulative number of new cases by municipality.

#### 2.3.2 Output flow

We assume that the production process is additive. Moreover, a worker that receives a daily pay of *w*_*i*_, generates

$$\begin{array}{c} o_{i}=\lambda w_{i} \end{array}$$ (10)

units of output per day. Hence, GDP is the sum of daily output throughout the year, of all workers. This technology entails that there are no dependencies among industries, that is, output in one industry depends solely on the workers who labor in it. This assumption is clearly restrictive; it has been used elsewhere where complementarities would appear to be a second-order concern, due to the time or spatial scale of the analysis ([19, 38]). In our case, it is a necessity in view of the data to which we have access. This assumption also entails that the wage bill is the same fraction of added value across industries. Hence, we measure (or rather approximate) output *o*_*t*_ as a factor *λ* of the sum of wages of the work that is actually done (either on-site, or remotely):

$$\begin{array}{c} o_{t}\equiv \lambda \sum_{i=1}^{n}w_{i}w_{it}. \end{array}$$ (11)

We track output as a fraction of the baseline: $\sum_{k=0}^{t}o_{k}/\left(to_{0}\right)$.

#### 2.3.3 Welfare

Ideally, one would wish for a measure of welfare: an all-encompassing variable that considers the suffering from human losses, from illness, from the stress and anguish of unemployment and hunger, as well as from the hardships of confinement. We don’t have that. Instead, we use a measure of material welfare that focuses solely on the urgent needs of the people who lose their income-generation capacity. We do this by means of a utilitarian utility function whose argument is daily consumption flow. This measure assumes that people do not have savings; for the wide majority of the Chilean population, the lack of savings is a natural assumption, as the income may be subsistence level only with nothing for saving. According to the last available Chilean survey of consumer finances [39], 66 percent of households do not hold financial assets, and 64 percent declares not having saved at all in the last 12 months. This lack of savings affects mostly the lower-income households, and these are the people most likely dependent on conditional work/income. The measure also assumes that people do not receive family or governmental help of any kind. As such, it should not be interpreted as reflecting actual welfare, even under the narrow focus on consumption, but as to the material welfare loss that would have realized should nothing had been done to alleviate deprivation. As such, it is a measure of the profoundness of the shock. Under the assumption that conditional earners lose their income under lockdown, total welfare flow is measured by the following social utility function:

$$\begin{array}{c} U_{t}=\sum_{i=1}^{n}\frac{h_{it}^{1-\gamma }}{1-\gamma }, \end{array}$$ (12)

where $h_{it}=w_{it}\left(1-c_{i}\left(1-w_{i}\right)\right)$ is the daily labor income perceived by a worker, and *γ* ∈ (0, 1) is a parameter that affects the concavity of the utility function. Notice that a worker fails to receive her wage if her pay is conditional and she is unable to work.

We track the utility level per day, as a fraction of the baseline utility level, namely $\sum_{k=0}^{t}U_{k}/\left(tU_{0}\right)$.

## 3 Empirical implementation

We adopt a Monte Carlo simulation approach, and implement it on the Santiago Metropolitan Region (SMR). The region holds Chile’s capital as well as a few rural areas, with an estimated 8.1 million inhabitants in total, about half the country’s population, spread across 52 municipalities. We worked with 51/52 municipalities because one of them, *Alhué*, is not represented in *ENE*. Table 1 presents some descriptive statistics.

**Table 1:** Santiago Metropolitan Region descriptive statistics.

	SMR	Greater Santiago
Population	8.1 million	7.1 million
Workers	5 million	4 million
Municipalities	52	37

**Table 2:** Different estimates of teleworking ability and essentiality.

A. USA			
	remote	on-site	
essential^a^	30	37	67
non-essential	14	19	33
	44	56	
Source: [51].			
B. Chile’s SMR			
	remote	on-site	
essential^b^	12	27	39
non-essential	21	40	61
	33	67	
Source: authors’ estimates.			

Notes:

^(*a*)^ Italian definition.

^(*b*)^ Chilean definition.

We draw from the following sources:

**ENE.** From Chile’s national statistics institute (*Instituto Nacional de Estadísticas*, acronym *INE*), we obtained the National Employment Survey (*Encuesta Nacional de Empleo, ENE*), December 2019 [40]. This fundamental document enabled us to obtain the following variables: municipality of residence and of work, employment situation (employed/unemployed), occupation (manager, professional, technician, machine operator, etc.), ISIC section (commerce, mining, agriculture, etc.), gender, age, and years of schooling. The full list is in S1. Table in S1 File. The number of observations is 19,584.
**ESI.** Also from *INE*, the Supplementary Income Survey (*Encuesta Suplementaria de Ingresos, ESI*), 2018 [41]. We obtained labor income and matched it by means of comparing up to ten categorical variables common to both surveys, to the individuals in *ENE*. S1 Appendix in S1 File details the procedure.
**CMI.** From Chile’s Ministry of Interior (*Ministerio del Interior, MI*), we obtained the national lockdown rules [42].
**SII.** From the Chilean internal revenue service (*Servicio de Impuestos Internos, SII*), we obtained firm statistics by municipality and economic activity, December 2018 [43]. Together with *CMI*, they are used to construct the probability of each worker type being under confinement.
**ER.** Chile’s Health Ministry (*Ministerio de Salud, MS*) supplied weekly epidemiological reports, numbers 1 to 39 [44].
**C.** From *INE*, the 2017 census provided the total population by municipality [45, 46] the population projections.

The data were combined to produce a set of agent types, which in turn were expanded through cloning by means of the employment survey’s expansion factors, in the scale 1:15. The total number of agents is then 540,445. The simulation follows the agents’ health and labor states. The input variables are listed in S1. Table in S1 File. With them, we built an indicator of working in an essential sector or not ($e_{i}$); an indicator of being able to work from home or not ($z_{i}$); a wage (*w*_*i*_); and a wage conditionality indicator ($c_{i}$). The details are in the S1 Appendix in S1 File.

Out of the constructed variables, the ability to work from home and the quality of being essential are the most important. Regarding the former, there are many estimates around the globe [47, 48], and a handful for Chile. For instance, [49] use survey data related to physical and social factors as well as general types of job behaviors. These estimates find that overall, in the US, about 37 percent of jobs can be done at home. They also find considerable heterogeneity among metropolitan areas, ranging from 28 percent to 51 percent. Our own estimates, based on an ad-hoc classification from employment survey data, is that in the SMR the figure is 33 percent. [50], using [49]’s methodology on household surveys find that for Chile, the overall figure is 27 percent. Considering that Santiago has a disproportionate fraction of white-collar jobs, these estimates appear to be accurate.

Estimates of the fraction of essential workers are subject to the differing definitions of essentiality, besides the structural differences in economic activity among countries. [51] used the Italian definition to study US data, which lacks an official definition. Table 2 shows their estimated joint distribution of essentiality and remoteness ability (Panel A), as well as our own estimates for SMR (Panel B). A general finding in the literature is that the poorer the country, the lower the fraction of workers that can work from home.

### 3.1 Baseline

**Fig 3:**
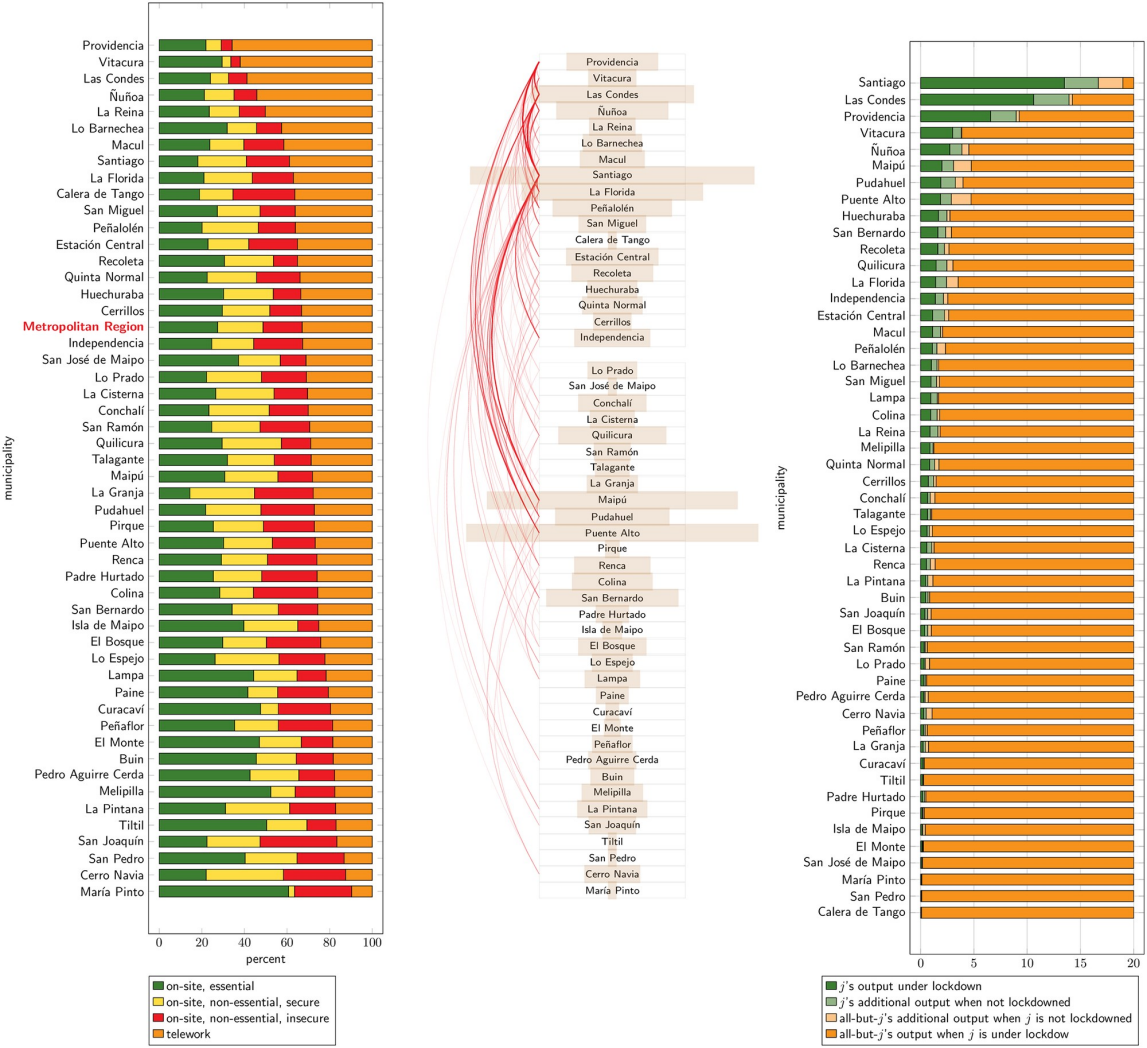
Baseline by municipality. (a) Percentage of employed workers by municipality of residence. Type I green; Type IIa yellow; Type IIb red; Type II orange. Sorted by the latter category. (b) In red, baseline commuting flows. The strongest connection is that of Providencia and Santiago; 1 percent of the SMR’s commuters travel between them. Only the strongest connections are depicted. Brown bars measure municipality size, by number of workers that live in each municipality. (c) Total output of each municipality (green) and of the rest of the SMR (orange), as a fraction of the latter. In light green, the decrease in the municipality’s output when it is put under lockdown. In light orange, the decrease in the rest of the SMR’s output when each municipality is put under lockdown.

Fig 3a depicts the composition of the set of employed workers by municipality, split in the four types defined in Section 2. The fraction of type-III workers varies widely among municipalities, ranging from 10 to 66 percent of the resident workforce. Type-I workers also varies widely among municipalities, ranging from 14 to 61 percent. The size of the type-I group sets a lower bound on mobility, as the size of the type-III group sets an upper bound. A lockdown doesn’t hinge upon the mobility of either type I or IV, but only on the other types. Thus, type IIa and IIb workers are the lockdown-sensitive group and range from 9 to 66 percent of workers, depending on the municipality.

Fig 3b shows labor-related commuting patterns. The strength of connections between municipalities is conjectured to be of the upmost importance in the initial stages of the transmission of the disease. The brown bars represent size, as they are proportional to the fraction of workers that live in each municipality. Puente Alto, Santiago, and Maipú, are the most populous municipalities.

If a municipality is under lockdown, the lockdown-sensitive workers cannot work and therefore, the output they generate is lost. However, many workers do work in a municipality other than their residences, which means that output will be lost not only in the municipality under lockdown, but also elsewhere. This effect has been noted previously; for instance, [52] estimate the lockdown effect in Tokyo to be twice as large in the rest of the country as in Tokyo itself. Fig 3c depicts the fraction of total SMR’s output for which each municipality is responsible (in green, dark and light); the output loss of each municipality if it is put under lockdown (in light green); the output of all other municipalities (in orange, light and dark); and the output of all other municipalities that would be lost if municipality *j* is put under lockdown (in light orange).

Several facts of interest are apparent in Fig 3c. First, the size distribution of output is far from uniform; Santiago, the largest municipality in this regard, is responsible for as much as 16.7 percent of the region’s output. Secondly, the effect of a municipality’s lockdown can be even larger on others than on itself. For instance, Maipú and Puente Alto, the largest municipalities by population and mostly residential in nature, as can be appreciated in Fig 3b, with roughly one third of their workforce being sensitive (Fig 3a), affect the rest of the region more than themselves when put under lockdown.

**Fig 4:**
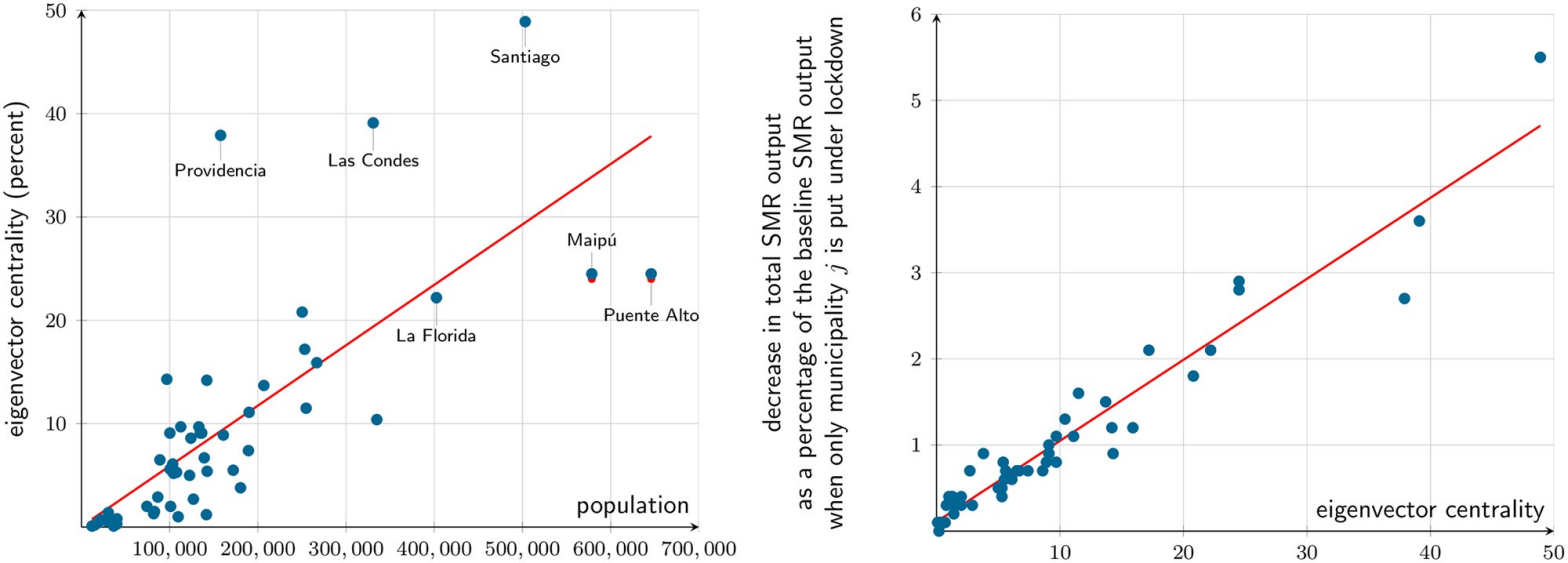
Eigenvector (commuting) centrality. (a) Eigenvector centrality vs. population. (b) Eigenvector centrality vs. municipal marginal output. Source: authors’ computations. Eigenvector centrality is computed from the commuting origin-destination matrix obtained from ENE [40], December 2019 (i.e., under no lockdown). Population, from 2020 projections [46].

Fig 4 further characterizes the commuting patterns in the SMR. We compute eigenvector centrality (defined as the eigenvector associated to the largest eigenvalue of the weighted adjacency matrix of the graph) as implied by the commuting origin-destination matrix obtained from ENE. Fig 4a relates it to population. Although there is a clear positive correlation between these two variables, a linear regression doesn’t provide a good fit. Indeed, according to the regression, the large residential municipalities referred to in the previous paragraph display low centrality for their size, while the more commercial municipalities, like Santiago, Providencia and Las Condes, lie far above the regression. On the other hand, Fig 4b relates it to the municipality marginal effect, that is, the total output loss to the whole of the SMR should each municipality–and only it–be put under lockdown. In this case, a linear relationship provides a much better fit: eigenvector centrality is closely related to the output loss from putting a single municipality under lockdown.

### 3.2 Calibration

The model has the following parameters: 〈*β*, *π*_*l*_, *κ*_1_, *κ*_2_, *π*_*d*_, *π*_*g*_, *n*_0_〉, namely, contagion parameter, isolation probability, stringent and non-stringent confinement parameters, death probability, detection probability, and characteristic population size for villages, respectively. We choose the first three from a grid based on the model fit, while *κ*_2_, *π*_*d*_, and *n*_0_ are set from the outside (to be explained shortly). In turn, the detection probability *π*_*g*_ is set temporarily to 1 to simulate the model, and selected afterwards to minimize the square distance between the observed and simulated number of cumulative cases for the whole SMR:

$$\begin{array}{c} \pi_{g}=\arg\min_{x\in [0,1]}\sum_{j}\sum_{t}{\left(i_{jt}^{w}-x{\hat{i}}_{jt}^{w}\right)}^{2}. \end{array}$$ (13)

Indeed, the official data [44] always report detected cases. Our simulation thus intends to reproduce the shape of the cumulative-case curve, and the detection probability adjusts its level.

For the purposes of the contagion process, we consider urban municipalities as cities, and rural municipalities as villages. The characteristic population size for villages is set to *n*_0_ = 157,950, corresponding to the largest number of people present simultaneously in any rural municipality.

The probability of dying is set to *π*_*d*_ = 0.4%. This number is the infection fatality ratio (IFR), as approximated by the fraction of cumulative deaths over the total cumulative cases as of July 31, 2020, assuming a detection rate of 10%. By way of comparison, [53] estimate that in low-income young-skewed population countries the average IFR is 0.23 percent (0.14–0.42, CI 95%), while in high-income countries, with a large proportion of elderly people, 1.15 (0.78–1.79 CI 95%). The SMR population lies between these extremes.

The adjustment factor for home stayers is set to *κ*_2_ = 0.56. This is motivated by the Google COVID-19 Mobility Report [54, May 15 to June 15] that shows an average decline in grocery-and-pharmacy-related mobility of 44 percent. Home stayers still go out for essential-good shopping; confinement reduces contact, but not completely. We assume that by default, when a municipality is locked down (a policy that includes both, restrictions to economic activity and “stay-at-home” rules), people who stay at home are under soft confinement. The exceptions are the elder (over 75 years old, when they are under mandatory confinement by age), and the group of highly educated, over 50 years old who are assumed to self-confine at the end of March. In these latter cases, the agents remain strictly confined at home from the beginning of their (mandatory or self-imposed) confinement, regardless of spatial targeted lockdowns.

The calibration proceeds in two stages. We first simulate the model, obtaining 5 realizations for each parameter vector 〈*β*, *π*_*l*_, *κ*_1_〉 in the grid {0.01,0.02, …, 0.99, 1.00} × {0.05, 0.06, …, 0.90} × {0.05, 0.1, 0.15, 0.2}. We choose the top 50 parameter configurations by fit, as measured by the Nash-Sutcliffe Model Efficiency Coefficient (NSE) of the weekly new detected cases by municipality, that is

$$\begin{array}{c} \text{NSE}=1-\frac{\sum_{t}\sum_{j}{\left(i_{jt}^{w}-{\hat{i}}_{jt}^{w}\right)}^{2}}{\sum_{t}\sum_{j}{\left(i_{jt}^{w}-\sum_{t}\sum_{j}\frac{1}{T}\frac{1}{m}i_{jt}^{w}\right)}^{2}}. \end{array}$$ (14)

**Fig 5:**
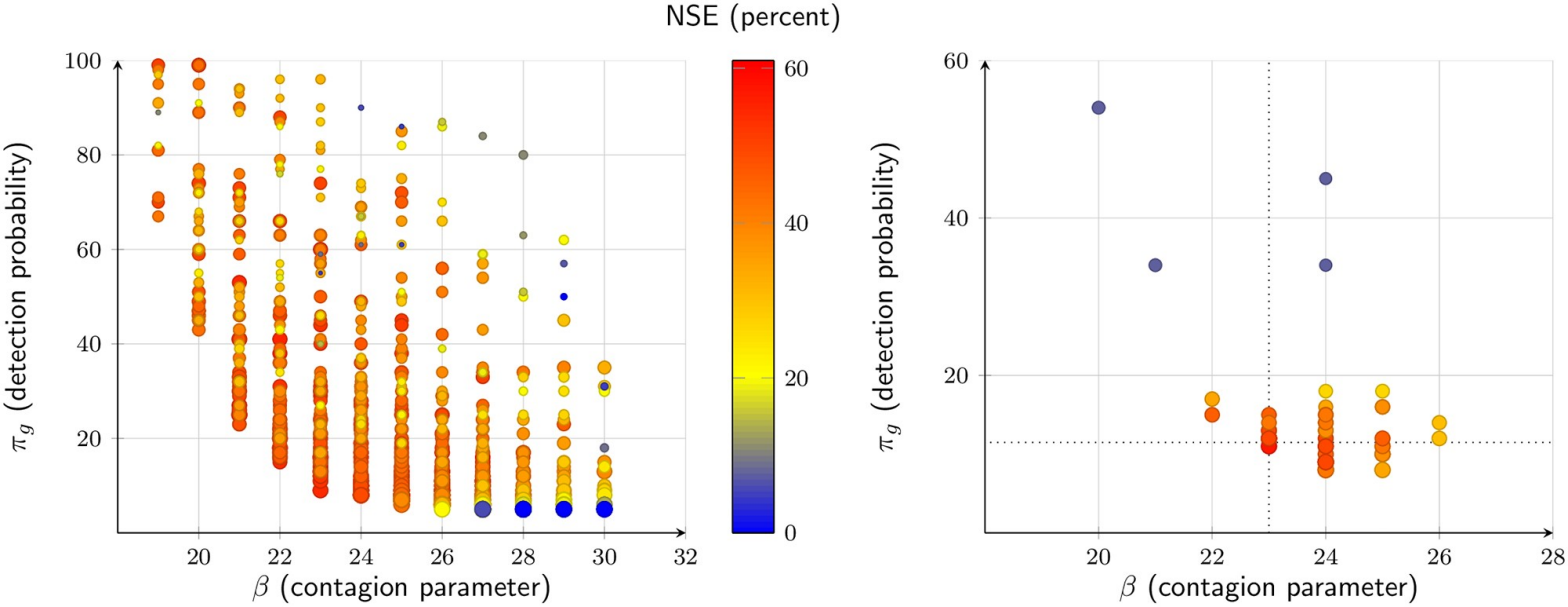
Parameter search. (a) First round. (b) Second round. Each dot corresponds to a simulation (5 realizations each in the first round, 100 realizations each in the second round). The top-50 paramater configurations made it to the second round. The color corresponds to the simulation’s Nash Sutcliffe Efficiency value on weekly new cases. Only realizations with positive NSE are depicted.

The second stage is to run 100-realization simulations on the selected parameter configurations and choose the best according to NSE. Fig 5 summarizes the NSE obtained in the first (Fig 5a) and second (Fig 5b) rounds, in the plane (*β*, *π*_*g*_). All simulations run from March 1, 2020, till August 1, 2020.

**Fig 6:**
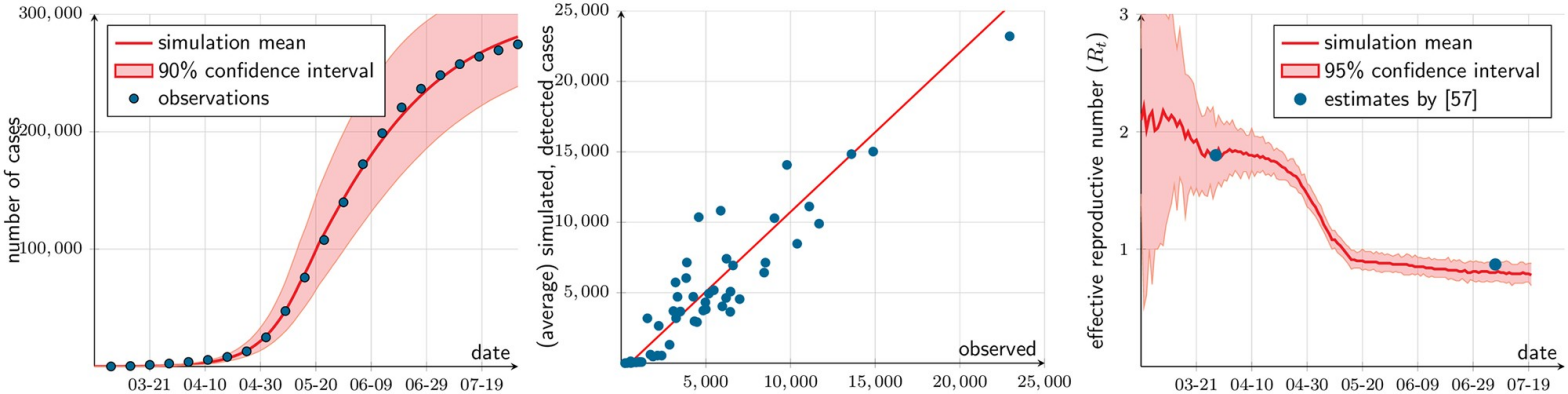
Model fit. (a) Cumulative detected cases: observed vs. simulated, March 1—August 1, 2020. (b) Cumulative detected cases by August 1, 2020, by municipality: observed vs. simulated. (c) Effective (case) reproductive number (*R*_*t*_).

The chosen parameter vector is *β* = 23%, *π*_*l*_ = 5%, and *κ*_1_ = 10%. The resulting detection probability is estimated at *π*_*g*_ = 11.49%. Table 3 presents summary statistics. Fig 6a depicts the actual cumulative number of cases for the Metropolitan Region in the first 22 weeks of the epidemic, as reported in the official weekly reports; the mean of 100 realizations run with the estimated parameters, as well as a the 5th and 95th percentiles of these realizations. The model fit is reasonable, reaching an NSE of 60 percent. The model also does a good job of explaining the municipality differences in infection rates, as can be seen in Fig 6b.

**Table 3:** Model fit as measured by the Nash-Sutcliffe Model Efficiency Coefficient.

Statistic	Value (percent)
NSE, weekly change in number of detected cases by municipality	60.08
NSE, cumulative number of detected cases by municipality	77.66
NSE, time distribution of weekly new cases, SMR	96.28

Some of our assumptions, in particular the lack of mobility types other than labor, make it difficult to compare the calibrated values of *β* and *π*_*l*_ with available empirical estimations. As an indirect verification, we compare the effective reproductive numbers *R*_*t*_ as defined by [55] and computed using [56]’s method, with the estimates by [57] for *R*_27_ (March 28) and *R*_128_ (July 7). Fig 6c shows that even though our simulation refers to the SMR and [57]’s to Chile, the estimates are quite consistent indeed.

The estimated detection probability seems low, for a country such as Chile that has done much testing. Still, the estimations for other regions or countries are not conclusive. For instance: [58] estimate that only 7.1% of the cases in China were reported at the beginning; [59] estimate for the United States 89% of undocumented cases until April 8 (i.e., a detection probability of 11.2%, with a 95% confidence interval of 4.9%—32.2%); finally, [60] estimate for Italy, Germany, Spain, UK, Greece, and Austria, until April 18, much higher detection rates (44.8%, 43.5%, 45.2%, 42.2%, 43.3%, and 41.7%, respectively). On the other hand, we assumed a constant probability of detection during the entire simulated time window and through all the spatial units, even though there was considerable variation in testing intensity across them.

**Fig 7:**
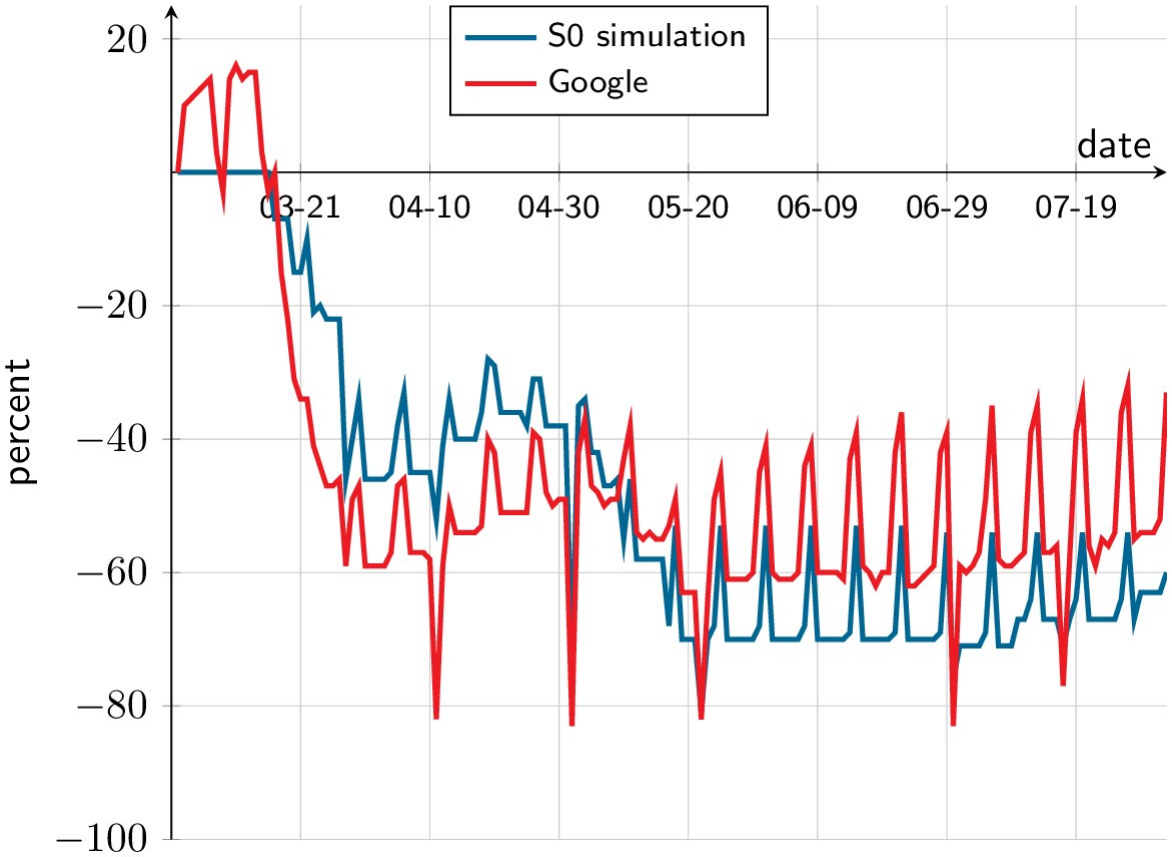
Mobility indices. The red line is Google’s labor-related mobility index, namely, the change in duration of stay in workplace with respect to same weekday of a reference week. The blue line is the same, as computed from simulations of the S0 scenario.

As a further check of the model fit, Fig 7 compares from Google’s COVID-19 Mobility Report [54, March 1 to August 1] the change in labor-related mobility in the SMR, with the model’s implied mobility. The resemblance is notorious, considering that Google’s index is built from the subset of the population that uses its location product while our index is based on the whole SMR population.

**Fig 8:**
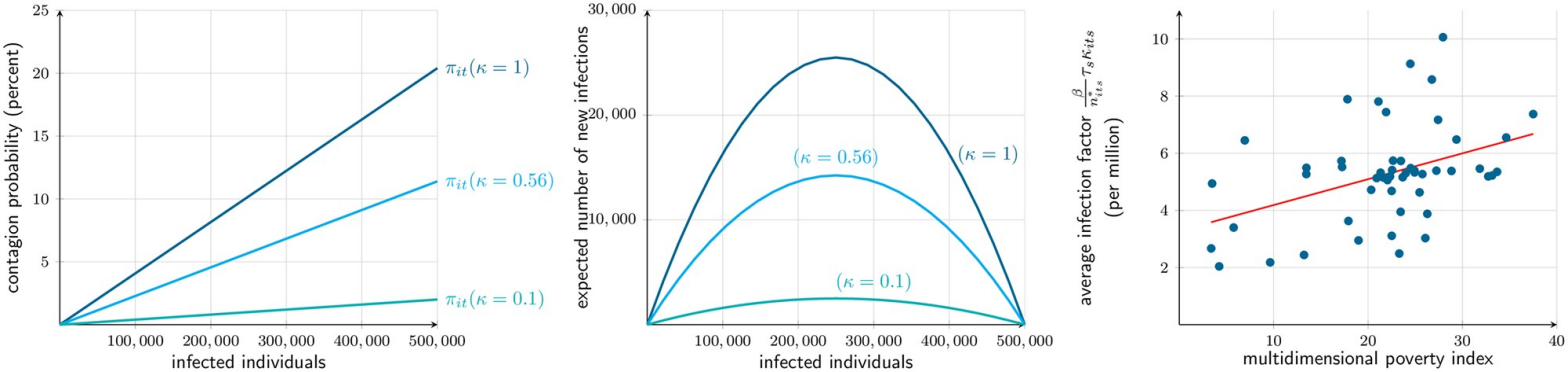
Estimated contagion probability in a municipality with 500,000 people present. (a) Daily contagion probability as a function of the number of infected individuals and the confinement degree. (b) Daily expected contagion flow as a function of the number of infected individuals and the confinement degree. (c) Estimated average infection factor for the residents of each municipality in the SMR at any labor business-day segment in the first two weeks of March, 2020, and multidimensional poverty index.

With the estimated parameters, the contagion probability *π*_*it*_ (Eq 6) becomes almost linear in the number of infected individuals in the population. Fig 8 illustrates it for the three degrees of confinement *κ*, assuming a municipality with half a million people present. Fig 8a illustrates the individual contagion probability. Although it runs from 0 to slightly above 20 percent, it should be noted that in simulations, rarely there will be a day with over 100,000 active cases in any municipality; hence, the “active” range is more in the order of 0 to 4 percent. Fig 8b depicts the expected number of new infections. The infection flow is almost quadratic on the number of infected individuals, as the flow is the product of the (almost linear) probability, applied to the non-infected individuals. The reduction in the new infection flow from lockdown is quite significant, as is the reduction from soft to hard confinement. Finally, Fig 8c shows that even though the model considers a common contagion parameter *β* across all municipalities, there are considerable differences among them in contagion factors, as defined by the product of all coefficients surrounding *β* in Eq 6. Moreover, these contagion factors do correlate with socio-economic conditions, like the multidimensional poverty index [61]: inhabitants of poorer municipalities are on average subject to higher health risk.

## 4 Results

We first ask how effective the implemented lockdown policy was. To answer this question, we compare the simulated trajectory of cumulative cases under the actual policy (scenario S0, or actual) with two extreme simulations: one without any lockdown (scenario S1, no lockdowns), and another that resembles scenario S0 until March 26, but considers a full lockdown since March 27 (scenario S2, full lockdown). The Chilean government implemented a spatial lockdown beginning March 27, affecting only seven municipalities. Before this date, it decreed school and shopping mall closures, among other closed activities.

**Fig 9:**
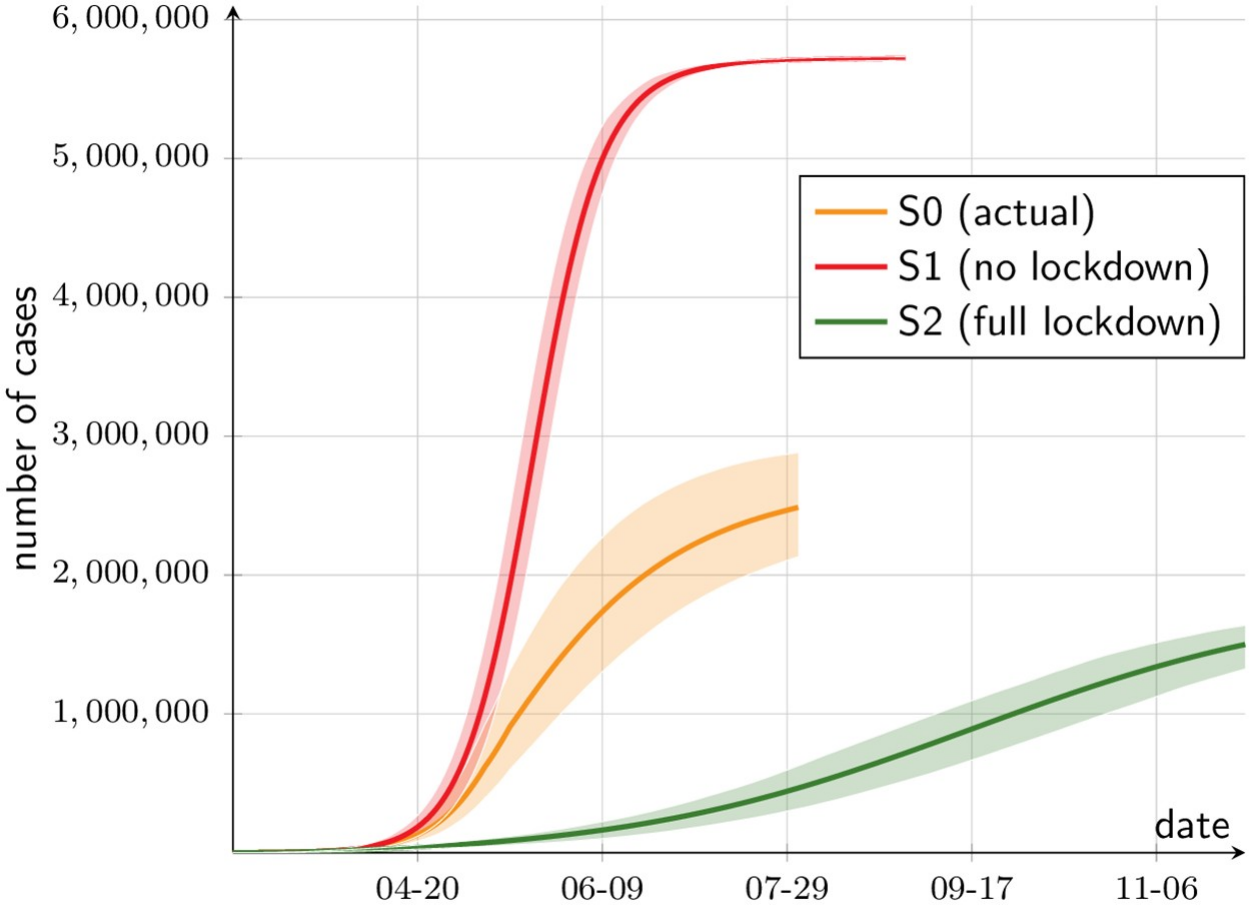
Cumulative cases in counterfactual scenarios. The lines depict the means across simulations, and the band around them the 90 percent confidence interval.

Hence, the question is what would have happened if, instead of locking down only seven municipalities and beginning a selective space-based lockdown, the government would have locked down all municipalities. Fig 9 shows the simulated trajectory for the whole of the SMR. In the counterfactual scenario S1, the disease simply spreads without control. Notice that the figure reports all cases, detected or not, so that the case numbers in S0 are 8.7-fold as high as the official figures, based on confirmed cases. Under no restrictions, S1 shows a quick evolution: in about four months herd immunity is reached with about 70 percent of the population having caught the disease at the end. On the other extreme, under full lockdown (S2), the total number of cases is a fifth of S0’s. In this sense, the actual lockdown policy (S0) proved effective, reducing the number of cases by 56 percent by August 1, 2020, as compared to S1. Yet, a 92 percent reduction from S1 would have been possible under full lockdown (S2).

**Fig 10:**
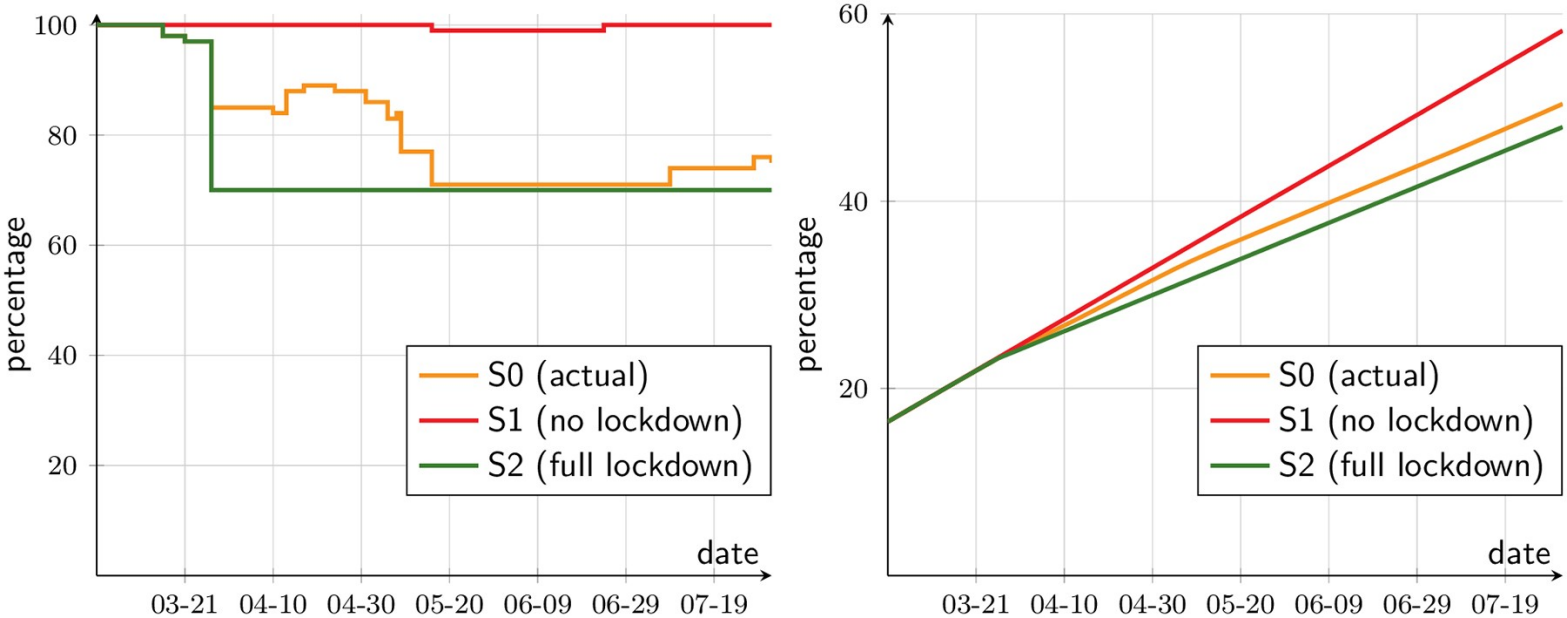
Output in counterfactual scenarios. (a) As a percentage of the baseline, daily, production. (b) As a percentage of the baseline, yearly, production.

Next, we look at the output loss. Since our data source is a labor survey, we approximate output as a multiple of the sum of the wages earned by all employed workers (within the SMR). How accurate this assumption is depends in part on how heterogeneous is the capital-labor intensity across industries. Fig 10 depicts output flows and cumulative output, as a percentage of the baseline. First, considering flows, the actual scenario (Fig 10a, orange line) exhibits considerable variability while the dynamic lockdown policy was in effect. Daily output falls to about 70 percent of baseline output, recovering slightly at the end. Without lockdown (scenario S1), the output drop is very small, and only lasts until workers are recovered from the disease. In contrast, under full lockdown (scenario S2) output stabilizes at 70 percent of the baseline.

Note that in this scenario, the fraction of output that is permanently lost, corresponding to those workers who died, is very small. Observe that the probability of dying was calibrated to the data, which reflects the mitigation policies and the probability of detection as well. Therefore, it does not consider the increase in mortality due to congestion and inability to care for overload in the health care system, a likely event in S1 scenario.

With the cumulative effect on output, the lines in Fig 10b are the integrals of the respective flows, starting January 1st. By August 1, 2020, 58.4 percent of the year has elapsed. In scenario S1 (no lockdown), 58.2 percent of baseline output has been produced. In the actual scenario (S0), 50.4 percent, that is, the estimated cumulative loss of five months of lockdown is 8 percent of GDP. Under full lockdown (scenario S2), output would have been 47.9 percent of the baseline, meaning that the cumulative loss would amount to 10.5 percent of GDP. This is to say, increasing the loss from 8 to 10.5 percent would have had the benefit of diminishing the number of cases, from 56 percent of S1, to 92 percent.

**Fig 11:**
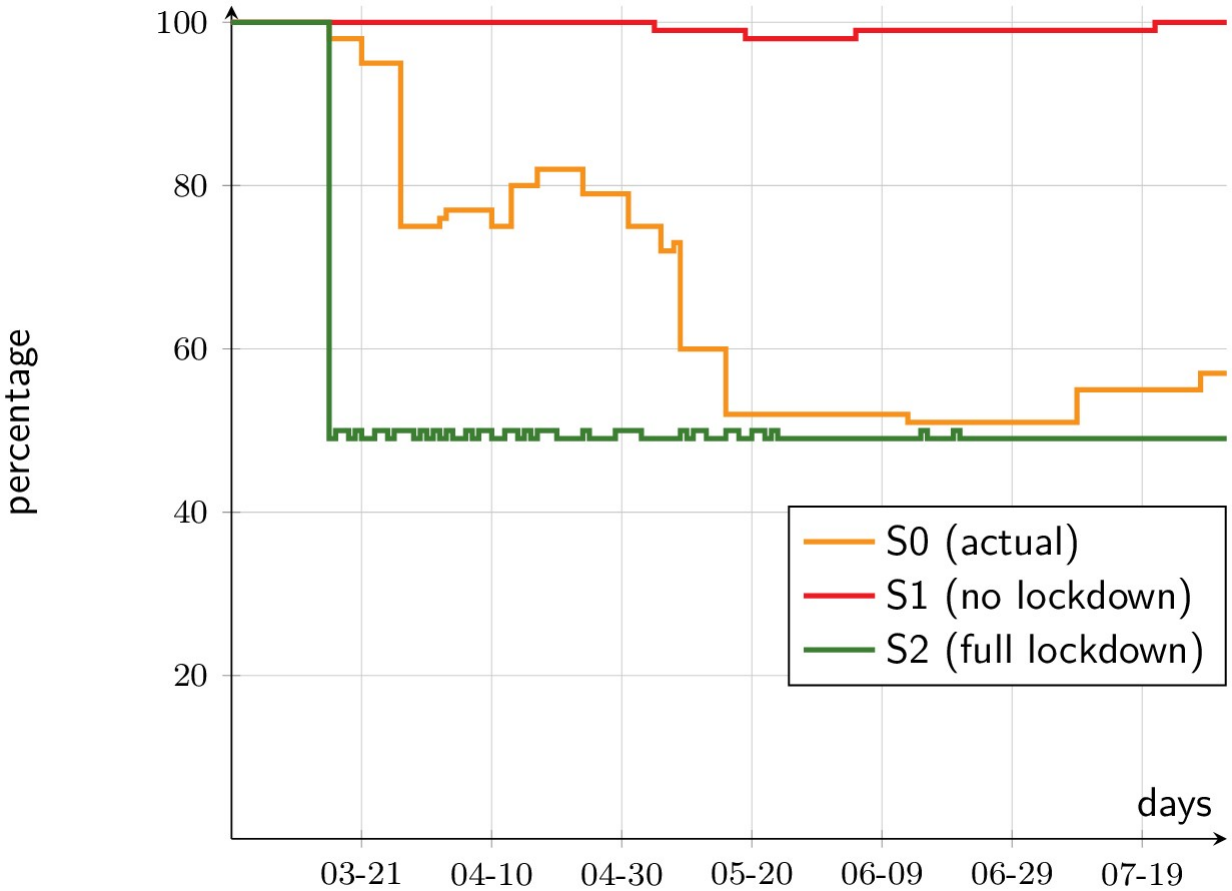
Material welfare index, without aid (percentage of baseline).

Fig 11 illustrates the eventual welfare outcomes should no aid package had been delivered, assuming a parameter *γ* = 0.75. Under full lockdown, the decrease in welfare is about 50 percent of the baseline. This is larger than the drop in output, because decreasing marginal utility makes the loss of vulnerable individuals more salient, in spite of the fact that there are workers who are paid even when not producing. Fig 11 is thus a testimony to how dire the situation would have been without aid packages of the sort we have seen in Chile, and worldwide, and shows how justified they were.

## 5 Further remarks

During this work, we have encountered several questions that call for further research. From a theoretical standpoint, we view metapopulation differential-equation models as mean-field-like approximations of agent-based models. Analytical models give us a fuller understanding of the processes under study, but usually demand more in the way of simplifications to maintain tractability. An important open question concerns the link between the two: How accurate an approximation is one to the other? We opted for an agent-based model in order to recover the parameters of a diffusion process from observations under constant regime changes. However, the question whether it is possible to estimate the process deep parameters in one model and use them in the other remains to be considered.

We used the model to assess the effectiveness of the pursued lockdown policy in SMR, as well as what it would have taken to get the maximum possible protection against the epidemic. The methodology developed in this paper, however, is amenable to the analysis of a vast array of complicated policies and can provide quick answers to questions such as to their effects on health, output, and welfare, with detailed information on the characteristics and location of the people involved. As such, this tool is not only useful to devise better lockdown policies but also helpful in pointing out where aid packages will be needed. Furthermore, this model can be applied to other disasters of natural origin such as earthquakes, floods, etc.

In addition, this model could be used to devise better, or more efficient policies. One advantage of a computational model over an analytical one is that it can more easily handle a large number of details that may prove relevant. For instance, some authors have argued in favor of time-varying stay-at-home policies [25] that affect different fractions of the population. The heterogeneity in labor compositions across municipalities may suggest that the restrictions by economic sector may be made municipality specific. Moreover, the very definition of what constitutes an essential activity could be made contingent on time and space, depending on the need.

## Supporting information

S1 File (ZIP)

S2 File Supporting materials: Code, input data files, and outcomes. DOI:10.5281/zenodo.4829304.
(ZIP)

## References

[1] Piguillem F, Shi L. Optimal COVID-19 quarantine and testing policies. Centre for Economic Policy Research; 2020;27:123–169.

[2] Agostini CA, Hojman D, Román A, Valenzuela L. Segregación residencial de ingresos en el Gran Santiago, 1992-2002: una estimación robusta. EURE Revista Latinoamericana de Estudios Urbano Regionales. 2016;42(127):159–184.

[3] Bro N, Mendoza M. Surname affinity in Santiago, Chile: A network-based approach that uncovers urban segregation. PLoS ONE. 2021;16(1):e0244372. doi: 10.1371/journal.pone.0244372 33406147 PMC7787389

[4] Romero H, Vásquez A, Fuentes C, Salgado M, Schmidt A, Banzhaf E. Assessing urban environmental segregation (UES). The case of Santiago de Chile. Ecological Indicators. 2012;23:76–87. doi: 10.1016/j.ecolind.2012.03.012

[5] Arino J, van den Driessche P. Disease spread in metapopulations. Fields Institute Communications. 2006;48:1–12.

[6] Keeling MJ, Rohani P. Modeling Infectious Diseases in Humans and Animals. New Jersey, USA: Princeton University Press; 2008.

[7] Brauer F, Castillo-Chavez C. Mathematical Models in Population Biology and Epidemiology. New York, USA: Springer; 2012.

[8] Hunter E, Mac Namee B, Kelleher J. A taxonomy for agent-based models in human infectious disease epidemiology. Journal of Artificial Societies and Social Simulation. 2017;20 (3)(2). doi: 10.18564/jasss.3414

[9] Eubank S, Guclu H, Kumar VSA, Marathe MV, Srinivasan A, Toroczkai Z, et al. Modelling disease outbreaks in realistic urban social networks. Nature. 2004;429:180–184. doi: 10.1038/nature02541 15141212

[10] Venkatramanan S, Lewis B, Chen J, Higdon D, Vullikanti A, Marathe M. Using data-driven agent-based models for forecasting emerging infectious diseases. Epidemics. 2018;22:43–49. doi: 10.1016/j.epidem.2017.02.010 28256420 PMC5568513

[11] Hunter E, Mac Namee B, Kelleher J. An open-data-driven agent-based model to simulate infectious disease outbreaks. PLoS ONE. 2018;13(12):e0208775. doi: 10.1371/journal.pone.0208775 30566424 PMC6300276

[12] Kuhl E. Data-driven modeling of COVID-19—Lessons learned. Extreme Mechanics Letters. 2020;40(100921). doi: 10.1016/j.eml.2020.100921 32837980 PMC7427559

[13] Aleta A, Moreno Y. Evaluation of the potential incidence of COVID-19 and effectiveness of containment measures in Spain: a data-driven approach. BMC Medicine. 2020;18(157). doi: 10.1186/s12916-020-01619-5 32456689 PMC7250661

[14] Calvetti D, Hoover A, Rose J, Somersalo E. Metapopulation network models for understanding, predicting, and managing the coronavirus disease COVID-19. Frontiers in Physics. 2020;8(261). doi: 10.3389/fphy.2020.00261

[15] Coletti P, Libin P, Petrof O, Willem L, Abrams S, Herzog SA, et al. A data-driven metapopulation model for the Belgian COVID-19 epidemic: assessing the impact of lockdown and exit strategies. Preprint at medRxiv. 2020. doi: 10.1101/2020.07.20.20157933 PMC8164894 34053446

[16] Costa GS, Cota W, Ferreira SC. Outbreak diversity in epidemic waves propagating through distinct geographical scales. Physical Review Research. 2020;2(043306). doi: 10.1103/PhysRevResearch.2.043306

[17] Hoertel N, Blachier M, Blanco C, Olfson M, Massetti M, Sánchez Rico M, et al. A stochastic agent-based model of the SARS-CoV-2 epidemic in France. Nature Medicine. 2020;26:1417–1421. doi: 10.1038/s41591-020-1129-4 32665655

[18] Gatto M, Bertuzzo E, Mari L, Miccoli S, Carraro L, Casagrandi R, et al. Spread and dynamics of the COVID-19 epidemic in Italy: Effects of emergency containment measures. Proceedings of the National Academy of Sciences. 2020;117(19):10484–10491. doi: 10.1073/pnas.2004978117 32327608 PMC7229754

[19] Alvarez FE, Argente D, Lippi F. A simple planning problem for COVID-19 lockdown. National Bureau of Economic Research; 2020. 26981.

[20] Birge JR, Candogan O, Feng Y. Controlling epidemic spread: Reducing economic losses with targeted closures. Becker Friedman Institute; 2020. 57.

[21] Fajgelbaum P, Khandelwal A, Kim W, Mantovani C, Schaal E. Optimal lockdown in a commuting network. National Bureau of Economic Research; 2020. 27441.

[22] Eichenbaum MS, Rebelo S, Trabandt M. The macroeconomics of epidemics. National Bureau of Economic Research; 2020. 26882.

[23] Farboodi M, Jarosch G, Shimer R. Internal and external effects of social distancing in a pandemics. National Bureau of Economic Research; 2020. 27059.

[24] Glover A, Heathcote J, Krueger D, Ríos-Rull JV. Health versus wealth: On the distributional effects of controlling a pandemic. National Bureau of Economic Research; 2020. 27046.

[25] Acemoglu D, Chernozhukov V, Werning I, Whinston MD. Optimal targeted lockdowns in a multi-group SIR model. National Bureau of Economic Research; 2020. 27102.

[26] Favero CA, Ichino A, Rustichini A. Restarting the economy while saving lives under COVID-19. Centre for Economic Policy Research. 2020. DP14664.

[27] Gollier C. Cost-benefit analysis of age-specific deconfinement strategies. Journal of Public Economic Theory. 2020;22(6):1746–1771. doi: 10.1111/jpet.12486

[28] Janiak A, Machado C, Turén J. COVID-19 contagion, economic activity and business reopening protocols. Journal of Economic Behavior and Organization. 2021;182:264–284. doi: 10.1016/j.jebo.2020.12.016 33390632 PMC7759096

[29] Asahi K, Undurraga E, Valdes R, Wagner R. The effect of COVID-19 on the economy: evidence from an early adopter of localized lockdowns. Journal of Global Health. 2021;11:05002. doi: 10.7189/jogh.10.05002 33643635 PMC7897430

[30] Fadinger H, Schymik J. The costs and benefits of home office during the COVID-19 pandemic: Evidence from infections and an input-output model for Germany. COVID Economics, Centre for Economic Policy Research. 2020;9:110–137.

[31] Ludvigson SC, Ma S, Ng S. COVID-19 and the macroeconomic effects of costly disasters. National Bureau of Economic Research; 2020. 26987.

[32] Colizza V, Pastor-Satorras R, Vespignani A. Reaction-diffusion processes and metapopulation models in heterogeneous networks. Nature Physics. 2007;3(4):276–282. doi: 10.1038/nphys560

[33] Colizza V, Vespignani A. Epidemic modeling in metapopulation systems with heterogeneous coupling pattern: Theory and simulations. Journal of Theoretical Biology. 2008;251(3):450–467. doi: 10.1016/j.jtbi.2007.11.028 18222487

[34] Lund H, Lizana L, Simonsen I. Effects of city-size heterogeneity on epidemic spreading in a metapopulation: A reaction-diffusion approach. Journal of Statistical Physics. 2013;151(1–2):367–382. doi: 10.1007/s10955-013-0690-3

[35] Belik V, Geisel T, Brockmann D. Recurrent host mobility in spatial epidemics: beyond reaction-diffusion. The European Physical Journal B. 2011;84(4):579–587. doi: 10.1140/epjb/e2011-20485-2

[36] Balcan D, Vespignani A. Phase transitions in contagion processes mediated by recurrent mobility patterns. Nature Physics. 2011;7(7):581–586. doi: 10.1038/nphys1944 21799702 PMC3142963

[37] Gómez-Gardeñes J, Soriano-Paños D, Arenas A. Critical regimes driven by recurrent mobility patterns of reaction–diffusion processes in networks. Nature Physics. 2018;14(4):391–395. doi: 10.1038/s41567-017-0022-7

[38] Nordhaus W. Geography and macroeconomics: New data and new findings. Proceedings of the National Academy of Sciences. 2005;103(10):3510–3517. doi: 10.1073/pnas.0509842103 PMC1363683 16473945

[39] Banco Central de Chile. Documento de Resultados EFH 2017; 2018. Available from: https://www.efhweb.cl/.

[40] *Instituto Nacional de Estadísticas* (National Statistics Institute), Chile. *Encuesta Nacional de Empleo (ENE)*; 2019.

[41] *Instituto Nacional de Estadísticas* (National Statistics Institute), Chile. *Encuesta Suplementaria de Ingresos (ESI)*; 2018.

[42] *Ministerio del Interior* (Ministry of Interior), Chile. *Instructivo para permisos de desplazamiento*; 2020.

[43] *Servicio de Impuestos Internos* (Internal Revenue Service), Chile. Estadísticas de Empresa; 2020.

[44] *Ministerio de Salud* (Ministry of Health), Chile. *Informe epidemiológico enfermedad por COVID-19* (Epidemiological report); 2020.

[45] *Instituto Nacional de Estadísticas* (National Statistics Institute), Chile. *Censo de Población y Vivienda*; 2017.

[46] *Instituto Nacional de Estadísticas* (National Statistics Institute), Chile. Proyecciones de Población; 2020.

[47] Gottlieb C, Grobovsek J, Poschke M, Saltiel F. Working from home in developing countries. European Economic Review. 2021; 133:103679. doi: 10.1016/j.euroecorev.2021.103679

[48] Lekfuangfu WN, Piyapromdee S, Porapakkarm P, Wasi N. On Covid-19: New implications of job task requirements and spouse’s occupational sorting. COVID Economics, Centre for Economic Policy Research. 2020;12:87–103.

[49] Dingel JI, Neiman B. How many jobs can be done at home? Journal of Public Economics. 2020;189:104235. doi: 10.1016/j.jpubeco.2020.104235 32834177 PMC7346841

[50] Delaporte I, Peña W. Working from home under COVID-19: Who is affected? Evidence from Latin American and Caribbean countries. COVID Economics, Centre for Economic Policy Research. 2020;14:200–229.

[51] Del Rio-Chanona RM, Mealy P, Pichler A, Lafond F, Farmer JD. Supply and demand shocks in the COVID-19 pandemic: An industry and occupation perspective. Oxford Review of Economic Policy. 2020;36(IS1):S94–S137. doi: 10.1093/oxrep/graa033 PMC7499761 40504218

[52] Inoue H, Todo Y. The propagation of economic impacts through supply chains: The case of a megacity lockdown to prevent the spread of COVID-19. PLoS ONE. 2020;15(9):e0239251. doi: 10.1371/journal.pone.0239251 32931506 PMC7491714

[53] Brazeau NF, Verity R, Jenks S, Fu H, Whittaker C, Winskill P, et al. COVID-19 infection fatality ratio estimates from seroprevalence. MRC Centre for Global Infectious Disease Analysis, Imperial College London; 2020. 34.

[54] *Google LLC*. *Google COVID-19 Community Mobility Reports*; 2020.

[55] Wallinga J, Teunis P. Different epidemic curves for severe acute respiratory syndrome reveal similar impacts of control measures. American Journal of Epidemiology. 2004;160(6):509–516. doi: 10.1093/aje/kwh255 15353409 PMC7110200

[56] Liu QH, Ajelli M, Aleta A, Merler S, Moreno Y, Vespignani A. Measurability of the epidemic reproduction number in data-driven contact networks. Proceedings of the National Academy of Sciences. 2018;115(50):12680–12685. doi: 10.1073/pnas.1811115115 PMC6294899 30463945

[57] Tariq A, Undurraga EA, Castillo Laborde C, Vogt-Geisse K, Luo R, Rothenberg R, et al. Transmission dynamics and control of COVID-19 in Chile, March-October, 2020. PLoS Neglected Tropical Diseases. 2021;15(1):e0009070. doi: 10.1371/journal.pntd.0009070 33481804 PMC7857594

[58] Maugeri A, Barchitta M, Battiato S, Agodi A. Estimation of unreported novel coronavirus (SARS-CoV-2) infections from reported deaths: A Susceptible–Exposed–Infectious–Recovered–Dead model. Journal of Clinical Medicine. 2020;9(5) (1350). doi: 10.3390/jcm9051350 PMC7291317 32380708

[59] Wu SL, Mertens AN, Crider YS, Nguyen A, Pokpongkiat NN, Djajadi S, et al. Substantial underestimation of SARS-CoV-2 infection in the United States. Nature Communications. 2020;11 (4507). doi: 10.1038/s41467-020-18272-4 32908126 PMC7481226

[60] Böhning D, Rocchetti I, Maruotti A, Holling H. Estimating the undetected infections in the Covid-19 outbreak by harnessing capture–recapture methods. International Journal of Infectious Diseases. 2020;97:197–201. doi: 10.1016/j.ijid.2020.06.009 32534143 PMC7286831

[61] *Ministerio de Desarrollo Social* (Ministry of Social Development), Chile. *Estimaciones de Pobreza Comunal 2017*; 2017.

