# Supplementary material for: Assessing the short-run effects of lockdown policies on economic activity, with an application to the Santiago Metropolitan Region, Chile
Source: PLoS One. 2021 Jun 21;16(6):e0252938. doi: 10.1371/journal.pone.0252938 (PMC8216525; doi:10.1371/journal.pone.0252938)
Supplement: S1 File — (ZIP) [file pone.0252938.s001.zip › 7.SI.pdf]

Supplemental Information for
Assessing the short-run effects of lockdown policies on economic activity, with an application to the Santiago Metropolitan Region, Chile

Constanza Fosco[1,2], Felipe Zurita[1,3 *]

1 Research Center for Integrated Disaster Risk Management (CIGIDEN), ANID/FONDAP/15110017, Santiago, Chile
2 *Grupo Interdisciplinar de Sistemas Complejos (GISC)*, Spain
3 *Instituto de Economía, Pontificia Universidad Católica de Chile*, Santiago, Chile

* Corresponding author
E-mail: fzurita@uc.cl (FZ)

S1 Appendix. Variables.
Table S1 describes the variables taken from *ENE* [40]. We now explain how we constructed the job essentiality, teleworking ability, wage, and wage security variables.

Essential activities. For each observed worker, *ENE* [40] reports the International Standard Industrial Classification code (ISIC, revision 4), at the section level (variable `ISIC section` in Table S1). Hence, we know if a particular agent works in manufacturing, but we do not know if she works in "manufacturing of food products" or in "manufacturing of jewelry, trinkets, and related articles," for instance. The difference is important, because lockdown rules state that the first kind of manufacturing activities are essential, while the second are not. To overcome this problem, we proceed as follows.

We attach to each essential activity described in the lockdown rules [42] the closest set of codes at a finer granularity than ISIC section (see Table S2). Next, using the *SII* firm statistic records by municipality and economic activity [43], we compute the number (plus one to avoid indefiniteness) of workers employed in firms that produce essential goods or services. In the case of "restaurants and food delivery" (code 561000, section "accommodation and food service activities"), we consider 50% of workers. Finally, we compute for each municipality and ISIC section the ratio of workers in essential activities over total workers. We assume that this ratio (by municipality and ISIC section) is the probability that an individual is allowed to work under lockdown.

We consider some special situations. First, if the worker commutes outside SMR, we assume that she is able to commute if and only if she works for a formal firm, regardless of her kind of activity. Second, if the worker's job category is "indoor domestic service", we assume that she will be able to work by staying at her workplace all the time. Finally, if the individual works for another household but not providing indoor domestic services, we set the probability to 0, regardless of her activity.

Until August 1, 2020, the health authorities issued nine different set of rules. Before the lockdown policy was in place, some activities were banned or restricted throughout the country. Specifically: (i) from March 16 on, all the face-to-face educational activities were banned (this affects section **16**, all codes); (ii) from March 19 onward, all public servants able to work from home were allowed to do it (section **15**, excluding high hierarchy officers); (iii) from March 19 onward, big shopping malls were closed (**7**, 471910); (iv) from March 21 onward, restaurants,

Table S1. Variables from the labor survey *ENE* [40]

Code name	Type	Description	Range
`ident`	static	id-matching to the correspondent survey observation	$\{0;1;\ldots;19,584\}$
`gender`	static	gender	{1,2}:{male, female}
`activ`	static	labor status	$\{0,1,2,3\}$:{children, employed, unemployed, inactive (adult)}
`age`	static	age range	$\{1,2,\ldots,17\}:\{[0,4],[5,9],[10,14],\ldots,[75,79],[80,\ldots)\}$
`age2`	static	age range (coarsening of `age`)	$\{1,2,3\}:\{[0,14],[15,65],[66,\infty)\}$
`educ`	static	educational level	$\{1,2,3,4,5,99\}$:{very low, low, medium, high, very high, unknown}
`comm`	static	type of commuter	$\{0,1,2,3\}$:{non-commuter, intra-municipality, inter-municipality, outside SMR}.
`jornada`	static	workday	{1,2}:{full-time, part-time}
`jobcat`	static	job category	$\{1,2,\ldots7\}$: {employer, self-employer or own account worker, private sector employee, public sector employee, outdoor domestic service, indoor domestic service, family workers}
`ISIC section` (*rama*)	static	Standard Industrial Classification (ISIC, revision 4) code, section level	$\{1,\ldots,21\}$:{agriculture, forestry and fishing; mining and quarrying; manufacturing; electricity, gas, steam and air conditioning supply; water supply, sewerage, waste management and remediation activities; construction; wholesale and retail trade, repair of motor vehicles and motorcycles; transportation and storage; accommodation and food service activities; information and communication; financial and insurance activities; real estate activities; professional, scientific and technical activities; administrative and support service activities; public administration and defense, compulsory social security; education; human health and social work activities; arts, entertainment and recreation; other service activities; activities of households as employers; activities of extraterritorial organizations and bodies}
`sector`	static	tax-formality of the employer	$\{1,2,3\}$:{formal, informal, other household as employer}
`telew`	static	worker is able to work from home (model equivalent to $\mathbb{z}_i$)	$\{0,1\}$:{no, yes}
`place`	static	the physical place where the individual usually works	$\{1,2,3,4,5,6\}$:{office; other home; own home; outdoor public spaces and streets; construction sites, mines, farms; other places}
`group`	static	The international classification of her/his occupation based on ISCO-08	$\{1,\ldots,10\}$:{managers; professionals; technicians and associate professionals; clerical support workers; service and sales workers; skilled agricultural, forestry and fishery workers; craft and related trades workers; plant and machine operators and assemblers; elementary occupations; non identified}
`job formalit`	static	job formality	$\{1,2,3\}$:{formal, informal, not applicable}
`wage`	static	daily wage, in CLP	$\mathbb{R}_+$

pubs, and all kind of public entertainment activities were banned (**9**: 561000 (50%), 551001-3/9, 552000, 559001/9, 562900; and **18**: 900004, 910100/200/300.). Table S2 lists all the activities that are allowed under municipality lockdown, either total or partial.

Teleworking ability. We conjecture that the possibility of working remotely depends on the following *ENE* variables: `place`, `jobcat`, `group`, and `ISIC section`, as defined in Table S1. The binary variable $\mathbb{z}_i$ assigns the value 1 to the worker who is able to work remotely, as detailed in Table S3.

Table S2. Essential activities and ISIC codes

Essential activity	Sections and ISIC codes	Rules
Health institutions and laboratories, related activities (cleaning, food). Foster homes, nursing homes, etc.	**9**: 562900; **14**: 812100/901/909; **17**: all except 862020/032.	1 - 9
Pharmacies, chemical laboratories, chemical companies, drug producers.	**3**: 201, 202 (exc. 202200/901), 210, 325009; **7**: 466901, 477201.	1 - 9
Veterinary and bioterium services.	**13**: 750.	1 - 9
Animal keepers (zoos, stables, etc).	**18**: 910300, 931101.	2 - 9
Airport staff.	**8**: 522300.	1 - 9
Energy (all kind, all stages).	**4**: 351.	1 - 9
Water supply (all stages).	**5**: 360, 370; **8**: 493090.	1 - 9
Gas (LPG, portable/pipe; all stages).	**4**: 352; **7**: 477310.	1 - 9
Gas stations and fuel distributors.	**3**: 192; **7**: 466100, 473.	1 - 9
Highway staff.	**8**: 522130.	1 - 9
Telecommunications services, data center, etc.	**10**: 601-2, 611-3, 619-20, 631;**14**: 822.	1 - 9
Repair/maintenance of technological and computer systems.	**10**: 620900, 639900; **19**: 951100.	6 - 9
Banks, financial and insurance companies, security transport.	**8**: 801002; **11**: all; **14**: 829120.	1 - 9
Funeral services and cemeteries.	**19**: 960310, 960320	1 - 9
Garbage and recycling companies, septic tanks, and landfills.	**5**: 381-390	1 - 9
Postal service and delivery.	**8**: 531/2	1 - 9
Public administration and defense.	**15**: all.	1 - 9
Supermarkets, markets, supply centers, distribution and food production. Street fruit and vegetable markets. Local food, hardware, and basic supply stores, served by their owner.	**3**: 101-110 (exc. 110110/120); **7**: 463 (exc. 463030), 471100,472 (exc. 472200/300), 478; **9**: 561100 (50%, food delivery); **14**: 829200.	1 - 9
Agro-food, fishing, poultry and fish farming, animal food, cellulose, paper and derivatives production.	**1**: all; **3**: 170; **7**: 461 (exc.461009), 462.	1 - 9
Public transport. Transport of goods (storage, loading, and unloading).	**8**: 491, 492 (exc. 492210 /240 /250 /290), 501200,502200, 512, 521,522120/400.	1 - 9
Janitors and property security officials. Security companies.	**14**: 801/2	1 - 9
Journalists and information media.	**10**: 581300, 591 (exc.591400), 639100; **18**: 900004.	1 - 9
Hotels with guests.	**9**: 559001.	1 - 9
Mining, basic iron, and steel industries.	**2**: all; **3**: 241/3; **7**: 466200.	1 - 9
All kinds of online sales.	**7**: 479100	1 - 9
Notaries and real estate conservators.	**13**: 691002/4.	2 - 9
Foreign trade (customs agents).	**8**: 522910.	2 - 9
Maintenance, repair and operation of public infrastructure.	**6**: 422000, 429000; **14**: 811000.	2 - 9
Cargo companies, storage, maintenance and repair services for transport.	**8**: 522190/990.	3 - 9
Plumbers and electricians (accredited).	**6**: 432200, 432100.	9
Technical revision plants (revision of trucks and public transport).	**13**: 712001.	9
After-sales service of construction (real state residential use).	**6**: 433000.	9

Source: authors' classification.
Emergency services of any kind are essential; they are included in the codes of the corresponding activities (energy, gas, defense, etc).
We were not able to find representative codes for some activities such as "Worship ministers exclusively for rites or activities that cannot be postponed" or "Maintenance and repair of elevators".

Wage. Daily income is obtained from the Supplementary Income Survey (ESI) [41], and matched to the 12.2019-National Employment Survey (ENE) [40] using a set of variables in common. In particular, we impute every worker in *ENE* the average wage of workers in the

Table S3. Definition of teleworking ability, based on *ENE* variables

$\mathbb{z}_i = 1$ if and only if:
`place` $\in \{1,3\}$: works at an office or at own home (65% and 7.3%, respectively; i.e. 72.3% of total employed workers); *and*
`jobcat` $= 1$: is an employer (4.9% of workers in office/own home); *or*
`jobcat` $= 2$ *and* `group` $\in \{1,2,3,4,5\}$: is a self-employer manager, professional, technician, clerical support worker, or service and sales worker (8.7% of workers in office/own home); *or*
`jobcat` $\in \{3,4,7\}$: is a dependent worker (private and public sector, or family worker; 82.7% of workers in office/own home) *and*:
`group`$= 1$ and `ISIC section`$\neq 15$: is a manager, but doesn't work in the public sector (5.1% of dependent/family workers in office/own home); *or*
`group`$= 2$ and `ISIC section` $\notin \{1,2,4,5,17,20,21\}$: is a professional (16% of dependent/family workers in office/own home), *or*
`group`$= 3$ and `ISIC section` $= 15$: is a technician working in the public sector (2.8% of dependent/family workers in office/own home); *or*
`group`$= 4$ and `ISIC section` $\in \{3,6,10,12,13,16\}$: is a clerical support worker in manufacture, construction, communication, real state, professional institutions, education (2.2% of dependent/family workers in office/own home); *or*
`group`$= 5$ and `ISIC section` $\neq 7$: is a seller but does not work in commerce (12.5% of dependent/family workers in office/own home).
Total: 32.9% of the currently employed workers.

same category in *ESI*, where "category" is defined by the product of the following (ordered list of) variables: `age2`, `gender`, `jornada`, `jobcat`, `educ2`, `ISIC section`, `sector`, `work municipality`, `group`, and `job formality`.

Nevertheless, should a group formed by the product of these ten variables have less than 5 observations in *ESI*, the last variable in the list would be dropped. Similarly, should a group formed by the product of these nine variables have less than 5 observations in *ESI*, the last variable in the remaining list would be dropped, and so on, until the group has 5 or more observations.

The resulting wages were updated to March 2020 by means of the corresponding wage index, by section level [62].

Wage conditionality. The variable wage conditionality ($\mathbb{c}_i$) takes the value 1 if and only if the worker can't work in any mode (neither on-site nor remotely) but still receives her income. We assume this is the case with private and public sector dependent workers (`jobcat`$= 3,4$), the employer is in the formal sector (`sector`$= 1$), and the job is formal (`job formality`$= 1$).

S2 Appendix. The simulation model.

We enter in some specific details of the simulation model. We use as guidance the ODD protocol [63], but simplified and adapted to our exposition structure.

Model description. We simulate, on a daily and individual basis, the co-evolution of an epidemics (SIR-like) and the labor force activities (move, work, telework, etc.), modulated by the (environmental) dynamic enactment of different non-pharmaceutical interventions.

The model is data-driven, agent-based, and with a metapopulation spatial approach, and we implement it with the computer software Python 3.7.6, within the Anaconda open-source distribution package ([64]).

It is data-driven because we obtain the population of agents (and all their static characteristics) from the expanded National Employment Survey [40], scaled 1:15. We chose

this survey because it is the unique data source that contains labor commuting data at the municipality level. This information has no longer being included in the census since 2002. The population includes children, unemployed, employed, and inactive people.

It is agent-based because we consider each agent's exposition to the disease and mobility feasibility depending on her current space-time situation. For her exposition to the disease, we consider where (home/workplace), when (morning, afternoon, evening), and how (confined or not) the agent is; for her mobility feasibility, essentiality and telework ability.

It has a metapopulation spatial structure because we distribute the total population according to their actual municipality of residence (subpopulations, 51 municipalities) and connect the municipalities through the actual labor commuting flows. So far, we explicitly move the commuters according to their work shift and assume that the contagion process occurs within each municipality (under the assumption of well-mixed but effective population present at each relevant moment of the day).

The model was designed for a specific region (SMR), yet it could be adapted to other regions or countries (in particular when the ex-ante heterogeneity is large and/or the mitigation policy is targeted by zones).

Entities, state variables, and scales.

Agents. Agents are people. For those workers who commute (either within a subpopulation or between subpopulations), there are two agent's instances (the resident and its replica). The agents' variables are described in tables S1 and S4. Note that the name of the variable corresponds to the name in the simulation code.

The variables `wage`, `risk`, `group`, `place`, and `gender` are not loaded in the main code. Either because they are used ex-post for computing the patterns of production, and welfare (linking each agent to its variables' values through its survey representative observation ID, `ident`), or to create other variables.

Note that the labor status (`activ`) does not change over time. It corresponds to the initial status and then, we track in time if an initial employed worker is able to work or not through the variable `work`.

The variable values correspond to those associated to each survey observation. The exceptions are: `telew`, `pConf`, `wage`, and `risk` (`risk`$=1-$`wage conditionality`). The construction of these variables can be found below.

Along with `pConf`, agents working in commerce (ISIC section 7) have a specific pConf (`pConf7`) that corresponds to the probability of working on-site when the unique measure going on in both municipalities (residential/workplace) is the malls closure. During the time-horizon of our model, some municipalities were locked down for discontinuous periods of time. Between two lockdown periods, the commercial activity was almost fully operative, with the exception of malls. Hence, if a commuter works in that municipality and comes from a not-locked-down municipality, she will move with probability `pConf7`, but if she comes from a locked-down municipality she will move only if her activity is essential (with probability `pConf`).

Spatial units. A spatial unit is a SMR's municipality. There are 51 of 52 represented (we exclude a very small rural municipality, Alhué, because it is not represented in ENE). Each spatial unit has an id (`cut`), a set of 21 initial `pConf` (`confinrules`, by industrial section level –`ISIC section`) to be assigned to all workers whose workplaces are located in it, the specific rule for commerce (`confin7`, and a list of all agents' instances, residents, and replicas, who either live or work (or both) in there. The spatial units are the subpopulations of the metapopulation approach, and do not include any proper geographical component. Space is homogenous.

Table S4. Program-generated variables

Code name	Type	Description	Range
su	static	id of the spatial unit (municipality) where the agent (or replica) is located (residential for residents, workplaces' for replicas)	$\{0,1,...,50\}$
home	static	type of agent	{0,1}:{replica,resident}
on	dynamic	signals if the agent is currently present in su	{0,1}:{no,yes}
status	dynamic	health status	$\{0,1,2\}$:{susceptible, infected, removed}
day	dynamic	counter for days between infected, detected/isolated, removed.	$\{0,1,\ldots,14\}$
conf	dynamic	confinement situation	$\{0,1,2,21,3\}$:{not confined, municipality lockdown, activity closure, activity closure + municipality lockdown, confined by age and/or educational level}
isol	dynamic	isolation/mobility restriction	$\{0,1,2\}$:{not isolated, isolated when infected, banned mobility for elder commuters under mandatory confinement by age}
replica		for intra- and inter-commuters, points to the replica and viceversa.	
work	dynamic	is the employed currently working?	$\{0,1,2\}$={no, yes and at her workplace, yes from home (teleworking)}
risk	static	signals whether an employed worker is at risk of not of being paid if unable to work.	{0,1}:{isn't at risk, is at risk}
pConf	dynamic	(Exogenous) probability of belonging to an essential activity	real (0,1)

System of spatial units (SystemRM). The system is the metapopulation. It includes a list of spatial units and two data sets for its initialization.

Environment and scales. The environmental variables are time (`t`), weekday (`d`), scenario (`situation`). The time scale of the model goes as follows: each time step represents one day; each day in turn is partitioned in three periods (morning, afternoon, evening) when all the activity takes place. There is a last implicit period (night) where nothing happens. Each period lasts 6 hours, and the corresponding time scale is modulated by a factor that reduces the interaction time within each subpopulation. The factor is $6/24$ for morning, afternoon, and evening. The evening's factor is reduced to $5/24$ whenever the government decrees a military curfew for the nights (the code variables are `tau0` for morning and afternoon, `tau1` for evening). This partition is meant to distinguish between full-time and part-time workers, and to explicitly consider that the population (including those infected agents) varies during the day, provided that many workers commute (full-time workers spend two periods out of home and one period at home; part-time workers spend one period out of home and two periods at home). The exact day of the week is considered because on the weekends not all workers have to work (moreover, we distinguish between Saturdays and Sundays). We also consider five holidays included in the Chilean calendar in the time-horizon considered (from March 1 to August 1, i.e., 154 days). Three holidays are modeled as Saturdays, and two as Sundays.

The variable `situation` selects the scenario. In this paper we consider three scenarios: (i) S0 (`situation=0`) that corresponds to the "real" situation (so we include almost all the mitigation measures that were implemented through time); (ii) S1 (`situation=1`) that simulates the counterfactual of "not doing anything", with the exception of non-workers older than 65 years that self-confine from March 24 on; (iii) S2 (`situation=2`) that follows the real situation until March 26, and on March 27 (when in the real situation only seven municipalities

where locked down) we assume that a spatial uniform lockdown starts (i.e., imposed to the 51 municipalities). By setting the variable `situation` to any number different from 0, 1 or 2, the model runs without any intervention (or self-confinement behavior) at all.

We describe in Table S5 the scenario S0. The environmental variables are the following:

- **Municipality lockdown.** The lockdown encompasses two interventions: (i) residents must "stay-at-home", yet are allowed to go out for shopping, health care, etc. with a special online permit; (ii) only the essential economic activities are allowed. The essential activities are explicitly defined by the health authority and all those agents working in non-essential activities are not able to work on the job-site (independently of their residential location). The lockdown may be partial or total. A partial lockdown prescribes the geographical limits of its scope within the municipality territory. In the latter case, we estimate (using QGis) the fraction of population (agents/replicas) under the lockdown policy intersecting the geographical map of the lockdown (available as Product 29 in the MCTCI GitHub repository) with the 2017 census data at the block scale [65, 66].

- **Activity closure.** This closure is applied to all municipalities, and it banns or restricts some specific economic activity. It affects the labor mobility and the feasibility of working on-site.

- **Confinement by age**. It is a "stay-at-home" rule applied on some specific age group across all the municipalities.

- **Curfew.** We use this term to refer to a strict "stay-at-home" policy, imposed during the night (a military curfew). No one is allowed to go outside (only in exceptional situations).

- **Self-confinement**. This is not a mandatory confinement. We assume that at some point in time, some group (defined by age, education or labor status) decide to "stay-at-home" voluntarily (in a strict fashion).

- **Change of confinement rules.** The health authorities changed the definition of essential activities for some industrial sections.

Process overview and scheduling. The model is simulated in discrete time-steps that represent one day. Each day is further divided into three periods and their duration are directly included in the individual's contagion probability. Table S6 lists the subprocesses that take place at each time-step. A full description of the main subprocesses is included below.

The time-horizon for scenario S0 is from March 1 to August 1, 154 days. For scenario S1 and S2, the simulation lasted until August 31 (184 days) and November 30 (275 days), respectively.

The outcome of each scenario is obtained by means of a Monte Carlo simulation (100 realizations). At the end of each realization, all the dynamic variables are reset.

Design concepts

Emergence. The proper emergent outcome of this model is the epidemics unfold, conditional on the environmental changes (i.e., lockdown policies, activity closures, etc.) that may either restrict the mobility between municipalities or slow down the time of interaction of people confined at home.

Adaptation. We may consider as adaptive behavior the following agent-based rules: (i) when a worker is under lockdown and she is able to work from home (teleworking), she does; (ii) some workers decide to stay at home even if it is not mandatory (self-confinement); (iii) self-confined people and those confined by age reduce their interaction time by more than the rest of confined agents.

Table S5. Scenario S0 - environment

Date	Measure	Scope
March 1	first day (t=0)	
16	education closure	SMR, ind. sect. 16
19	remote working Publ.Adm.	SMR, ind. sect. 15
	malls closure	SMR, ind. sect. 7
21	entertainment events banned	SMR, ind. sect. 18
	sport facilities closure	SMR, ind. sect. 18
	restaurants, pubs closure	SMR, ind. sect. 9
22	curfew (night)	SMR
24	confinement by age	SMR, 80 years or more
	self-confinement	SMR, 50 - 79 years, high educ.,non-worker or able to work from home
27	lockdown	municip. 0, 7, 13, 14(97.3%), 19, 22, 31
	confinement rules issued (Instr. 1)	SMR, essential/non-essential activities
April 2	change conf. rules (Instr. 2)	SMR, ind. sect. 6, 8, 13, 14, 18
3	ending lockdown	municip. 7
10	lockdown	municip. 32(49.9%)
13	ending lockdown	municip. 14, 22, 31, 0, 19 (both from 100% to 76.8%)
17	ending lockdown	municip. 13
	lockdown	municip. 4, 38(42.1%)
	change conf. rules (Instr. 3)	SMR, ind. sect. 8
24	lockdown	municip. 7(29.7%), 20, 25
May 1	lockdown	municip. 5, 7(from 29.7% to 100%), 11(46.7%), 30(56.5%)
6	lockdown	municip. 0(from 76.8% to 100%), 1, 24(99.4%), 26
8	ending lockdown	municip. 19
9	lockdown	municip. 2, 3, 8, 9, 10, 11(from 46.7% to 100%), 15, 16, 17 , 21, 27, 28, 29, 30(from 56.5% to 100%), 32(from 49.9% to 97.7%) , 38(from 42.1% to 62.7%)
16	confinement by age	SMR, 75-79 years
	lockdown	municip. 6, 12, 13, 14, 18, 19, 22, 23, 24(from 99.4% to 100%), 31, 32(from 97.7% to 100%), 35, 36, 38(from 62.7% to 100%), 39, 49
27	change conf. rules (Instr. 6)	SMR, ind. sect. 10, 19
June 13	lockdown	municip. 34(34.3%), 37(27.7%), 42(48.4%), 43(57.4%), 50
27	lockdown	muncip. 40, 46, 47
July 9	change conf. rules (Instr. 9)	SMR, ind. sect. 6, 13
28	ending lockdown	municip. 12, 13, 14, 19, 31, 35, 37
	lockdown	municip. 48
August 1	last day (t=153)	

Interaction. The agents interact within each spatial unit, when they are either working or at home. As a typical metapopulation model, we assume a well-mixed population in the following sense: the fraction of infected/population is the same for all the agents that are present at that moment of the day at some specific spatial unit. But we allow some heterogeneity between spatial units and between three groups of agents within each spatial unit.

- (between spatial units) we distinguish between “cities” and “small villages” [34] so that in the “cities” the implicit average interaction time is reduced as the size of the population increases, while in the “small villages” it is constant (16 out of 51 municipalities are “small villages”);
- (between agents within a spatial unit) when there exist some lockdown measure and/or confinement rule by age, people at home reduce their interaction time, while workers at their workplaces do not; and from those staying at home, those confined by age (the elder)

Table S6. Scheduling

At each time-step (1 day):
•update environment (if any measure was implemented)
•update infected agents (isolation/detection, removal, infection-day)
•move commuters job category “indoor domestic service”
•update work status of workers who usually work at home
•move commuters (to workplace: full-time, part-time-morning, outside SMR)
•contagion, first round
•move commuters (back home: part-time-morning; to workplace: part-time-afternoon)
•contagion, second round
•move commuters (back home: full-time, part-time-afternoon, outside SMR)
•contagion, third round
•update from susceptible to infected (susceptible infected at least in one of the three rounds)
•update detected cases (only for calibration)
•create and save daily output files

or self-confined reduce their interaction time more than the rest.

Stochasticity. The model has the following elements that are stochastic:

- At the beginning of each realization, the part-time workers are randomly sorted (half and half) between morning and afternoon shifts.
- The number and municipality of residence of the initial infected agents are estimated from the real data, but they are chosen randomly among all the resident agents, for each realization.
- Within each contagion round, each susceptible agent can get the infection with some probability.
- The time-steps between getting infected and being removed are not stochastic, but after five days of being infected, each agent can be isolated with some probability. Furthermore, the last day, when the agent is removed, she may die with some probability.
- Under lockdown, a commuter who works in a partial essential activity (i.e. with $0 < \texttt{pConf} < 1$), and cannot telework, commutes with probability `pConf` each time-step (day).

Observation. For each time-step (day) and realization, we observe the distribution of agents of each type among the following combined nine compartments {not-working, working in-site, teleworking}x{susceptible, infected, removed}. The list of types is ordered by the variable `ident`, so that we are able ex post to link each agent with its characteristics and obtain many kinds of patterns. For instance, the compartments (health status, not working) include those agents who are not workers (children, unemployed, inactive), but by linking each row of the output file with the variable `activ`, we are able to consider only those initially employed that are not able to work. We also observe for each realization the final cumulative number of deaths (only this total, and for SMR). In the first scenario, we keep track of all the infected at their detection day (so that we can estimate the detection probability using the reported data), and the time spent at work (so that we can estimate the same measure reported by Google Analytics [54]).

Initialization. There are two different “initial conditions”. The first one concerns the initialization of the initial system of spatial units (data-driven). The second pertains to the initialization of each realization.

- We create a system (SMR) of 51 spatial units (municipalities). Each municipality is loaded with the agents/replicas according to the survey data, after pre-processing the data. This part requires two files. There are 19,584 types of agents, each type has a variable number of "clones". The number of residents in the model is 540,445, that together with the replicas of those workers who commute inside the region sum 785,794 Agent instances. The loading goes as follows: for each type, a certain number of residents are created (correspond to the scaled expansion factor), each of them with their static variables. If the resident is a commuter, a replica is created and both are linked by the agent variable `replica`. This system can be created before any simulation.

- The setup at the beginning of each realization is: (i) the initial `pConf` of each worker is assigned (it is initial in the sense that corresponds to the first set of rules that will be used according to the mitigation measures) (these were loaded to each spatial unit in the system initialization); (ii) initial infection cases: in our implementation, the number of initial cases for each municipality corresponds to the number of reported cases for the previous epidemiological week (ending at Feb. 29), assuming that only 5% where detected at that point and scaled by 15; thus the number is fixed (and specified inside the code as a list), but inside each municipality, the infected are randomly chosen at the beginning; (iii) random distribution of part-time workers between morning and afternoon shifts.

Input data. The main model needs two input files.

- A file with the initial `pConf` by municipality and ISIC section (*rama*) – those that are not trivial, i.e. not equal to 0 or 1 and `pConf7` (`data1`).

- A file with the 19,854 types of agents including the number of agents of each type and the associated variables values (`data2`).

For the calibration process two other files are used: (i) cumulative cases for SMR (by epidemiological week as reported in [44]), (ii) panel data by municipality and epidemiological week of cumulative cases (the code computes the increments).

Pre-processing: *ENE* [40] contains 19,854 observations for the SMR, including employed, unemployed, inactive and children. Each survey's observation has an individual expansion factor that is representative at the national and the regional levels. We transform this factor into a number of agents of our model with the same characteristics as the observed individual i. We obtain the distribution of age (using 17 intervals) conditional on the municipality of residence from *ENE* and from the population projected for 2020 by INE. We denote by $x_{i,j,k}$ the expansion factor that *ENE* attaches to the observation i, that corresponds to an individual who resides in the municipality j and whose age is in the interval k; $X_{j,k}$, the population residing in municipality j with age in the interval k according to *ENE* ($X_{j,k} = \sum_i x_{i,j,k}$); and $N_{j,k}$, the population residing in municipality j with age in the interval k according to the 2020 population projection. Then, the number of agents in our model identical to individual i (who resides in the municipality j and whose age belongs to the interval k) is

$$n_{i,j,k} = \left[\frac{x_{i,j,k}}{15} \frac{N_{j,k}}{X_{j,k}} \right] \tag{1}$$

where $\frac{1}{15}$ is the scale ratio. Each agent in our model represents 15 individuals of the projected population for the region, excluding Alhué. Since we have to round the number, the total sum is almost equal to the total population/15. The number of residents in the model is 540,445, that together with the replicas of those workers who commute inside the region sum 785,794 Agent instances.

Variable re-categorization: Age (grouped into 17 intervals), and educational level (reduced to 5 levels).

Data corrections: 26 observations (out of 19,584) did not have `ISIC section` (*rama*) recorded. We corrected them with the following criteria: if the job category reported was domestic service (indoor/outdoor, `jobcat` in $\{5, 6\}$) or if the place of work was "other household" (7 observations), we assigned them to ISIC section 20 (Activities of households as employers); otherwise, we assigned them to ISIC section 19 (Other service activities). These 26 observations correspond to 1436 out of 540,445 agents in our model (0.27%).

Subprocesses. Each time we refer to an event occurring with some probability (move, contagion, etc.), it means that the model draws a random number (uniform) and makes the usual comparison.

- **Update environment.** When a municipality is locked down, at the beginning of the day, all the agents are confined (the process sets `conf` = 1). If the lockdown is partial (say x% of the population), the model randomly selects x% of the agents and x% of the replicas and confine them. Note that for any commuter it can happen that the agent at home is under lockdown, but her replica (workplace) not, or vice versa. Although the effective behavior with respect to mobility would be the same, we have to distinguish because of the behavior at home. If an agent is already under some kind of confinement, the model proceeds as follows: when the agent is under activity closure (`conf`=2), it changes to (`conf`=21) to allow the possibility of going back to `conf`=2 in the case that the municipality lockdown ends, but the activity remained closed. When the agent is confined by age or self-confined (`conf`=3), her status does not change (`conf`=3 assumes more stringent and lasting stay-at-home behavior). When an activity is closed (like education at the beginning), all the agents and their replicas of the SRM working in that activity are selected and put under `conf`=2. Under mandatory confinement by age (elder agents), the agents (and their replicas) are put under `conf`=3 and, additionally, `isol`=2 to prevent that they move to work. When the health authority redefines the set of essential activities for some sector, the agent's `pConf` is updated. Finally, when the government decrees a curfew (at night), the model reduces the overall time of interaction in the third contagion round (i.e., `tau1`, from 6.0/24.0 to 5.0/24.0).
- **Update infected status.** Each infected agent has a counter for the days until being removed. This counter is updated until `day`=13, when the infected is randomly chosen to recover or to die. If the agent dies, her health status is set to 2, and is effectively removed from the system (`on`=0, `isol`=0). At `day`=6 the infected agent is randomly sorted between detected and/or isolated. Detection occurs (and matters only) during the simulation of scenario S0 with probability 1, because this feature is used for calibration and the detection probability is estimated ex-post. Isolation, instead, is modulated by a parameter, and means that the agent will be out of the system until she recovers (`on`=0, `isol`=0). When an isolated infected agent recovers, she is located back at home (`on`=1, `isol`=0). Naturally, all the health-related changes affect simultaneously the agent and its replica.
- **Move people working in "indoor domestic service".** These workers are assumed to work from Monday to Saturday. Therefore, they move Sundays from work to home, and return to work on Monday. In the case of municipality lockdown (either home or workplace), we assume that the agent stays at her workplace.
- **Check whether people usually working at home can still work.** Some workers work always at home (i.e. from the beginning, `comm`= 0). The method **does-Work** checks whether they are able to work or not an it is similar to the method that moves a commuter (see next item). It considers the possibility that the agent is under lockdown, her activity is not essential, and cannot telework (e.g., could be the case of a car mechanic who usually works at home). It returns the updated value of the variable work (working status).

- **Move commuters.** The model moves each commuter with the method **commute-toW** and returns her working status (variable work). It entails the following steps:
  - for agents working in `ISIC section`= 7 select the proper `pConf`
  - for commuters with replica (`comm` in $\{1,2\}$: if agent and replica are not confined (`conf`= 0), then move and set `work`= 1 (working on-site); otherwise, if the agent cannot telework and her job is non-essential (`telew`= 0 and `pConf`= 0), then set `work`= 0 (not working), if the agents cannot telework, but `pConf`> 0, move the agent with probability `pConf` (hence `work` might be 1 or 0), if the agent can telework (`telew`= 1), do not move, but set `work`= 2 (teleworking). Movement is modeled through the variable `on` (i.e. if an Agent instance is at home, Agent.on= 0, Agent.replica.on= 1 means a movement from home to work, and viceversa).
  - for commuters to outside SMR (`comm`= 3) the steps are similar, but there is no replica, so that the model simply sets `on`= 0 when the worker is at her workplace.

  When a commuter is not meant to go to work because it is Sunday, for example, the model checks with the method **does-Work** about whether this agent is entitled to work or not. The method **does-Work** follows the same steps as **commute-toW**, but doesn't move the agents (i.e., it simply updates the variable `work`, and performs the same randomization when the agent is confined and cannot telework).

- **Round of contagion.** At each round of contagion, the model computes the effective population currently present in the municipality, and the effective infected people that are not isolated (`on`=1, `isol`=0). For small villages, the population is constant, so that only the effective number of infected is counted. Then, for each susceptible agent currently present in the spatial unit, the model computes her individual probability of contagion and infects the agent with that probability. The probability of contagion is different for these three groups: people at home confined by rule `conf`=3, people at home confined by municipality lockdown (`conf` in $\{1,21\}$), and the rest (people at home not confined, and all workers at their workplaces). The difference is modulated by the parameters of confinement (`Ks`, `Kns` in the code). All the susceptible agents that get the infection at any of the three rounds are saved into a list of agents for updating at the end of the time-step.

- **Update the status of susceptible that get the infection during the day.** The health status of a susceptible agent that gets the infection is updated at the end. That is: the susceptible gets the disease if she is infected in at least one of the three rounds. The updating changes the health status and sets the day counter to 1.

- **Create and save the output file of the day.** Here, the model counts for each `ident` (i.e., agent type) how many agents are within each health and labor situation and delivers the daily output.

- **Data post-processing.** The output files are post-processed out of the main program.

References

62. *Instituto Nacional de Estadísticas* (National Statistics Institute), Chile. Nominal remuneration index (base 2016=100), National according to economic section (CIIU4.CL 2012), monthly; 2020.

63. Grimm V, Railsback SF, Vincenot CE, Berger U, Gallagher C, DeAngelis DL, et al. The ODD protocol for describing agent-based and other simulation models: A second update to improve clarity, replication, and structural realism. Journal of Artificial Societies and Social Simulation. 2020;23 (2)(7). doi:https://doi.org/10.18564/jasss.4259.

64. Anaconda Software Distribution; 2020. Available from: https://docs.anaconda.com/.

65. QGIS Development Team. QGIS Geographic Information System; 2020. Available from: http://qgis.org.

66. *Ministerio de Ciencia, Tecnología, Conocimiento, e Innovación* (Ministry of Science, Technology, Knowledge, and Innovation), Chile. *Datos COVID-19*; 2020.
